# One-Pot Total Synthesis of a Post-translationally Modified Max Transcription Factor Sheds Light on Ser-Phosphorylation and Lys-Acetylation Crosstalk in DNA Binding

**DOI:** 10.1021/acs.orglett.5c00978

**Published:** 2025-03-31

**Authors:** Raj V. Nithun, Shada Khoury, Muhammad Jbara

**Affiliations:** School of Chemistry, Raymond and Beverly Sackler Faculty of Exact Sciences, Tel Aviv University, Tel Aviv 69978, Israel

## Abstract

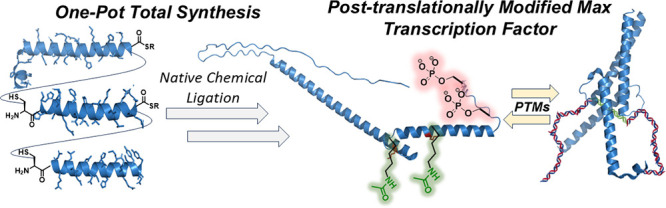

We report a one-pot total synthesis of the transcription factor Max in seven consecutive steps, starting from three peptide segments and employing native chemical ligation. The developed synthesis facilitates the generation of homogeneous Max analogues bearing defined transformations within hours in excellent yields, enabling us to probe the effect of the crosstalk between Ser-phosphorylation and Lys-acetylation on the Max function. Our findings reveal that these post-translational modifications significantly inhibit DNA-binding activity, potentially by disrupting essential Max–DNA interactions.

Protein post-translational modifications (PTMs) are essential for expanding proteome diversity by altering the protein structure, function, and cellular localization.^1^ Of particular interest are the transcription factor (TF) proteins, which regulate gene transcription by interacting with specific DNA sites to regulate gene expression.^2^ Their function is controlled by various mechanisms, including PTMs such as acetylation, phosphorylation, and methylation. For example, Max TF is regulated by PTMs at different strategic sites, which influence its DNA-binding activity and overall function.^3,4^ Max regulates gene expression by forming specific dimeric complexes, e.g., Myc/Max and Max/Max (Figure 1). This dimerization enables the recognition of the enhancer box DNA sequence (E-box), leading to either activation (Myc/Max) or repression (Max/Max) of gene expression. Max complexes regulate approximately 15% of the human genome and play crucial roles in cell growth, differentiation, and proliferation. Their dysregulation is implicated in nearly 70% of human cancers.^5^ Importantly, Max has been reported to undergo PTMs, e.g., acetylation and phosphorylation, which modulate its function. For instance, phosphorylation of Max at Ser-2 and Ser-11 has been shown to inhibit its DNA-binding,^6^ whereas acetylation at Lys-31 and Lys-57 modulates its DNA-binding affinity and sequence specificity.^7^ Despite progress in understanding the molecular role of the Lys-acetylation and Ser-phosphorylation patterns in Max TF, the potential crosstalk between these two essential modifications and its impact on Max–DNA binding and regulation remains unclear. A major challenge in gaining insight into PTM’s impact on the regulation of TF–DNA interactions lies in the difficulty of generating homogeneously modified TFs using conventional biological methods.

**Figure 1:**
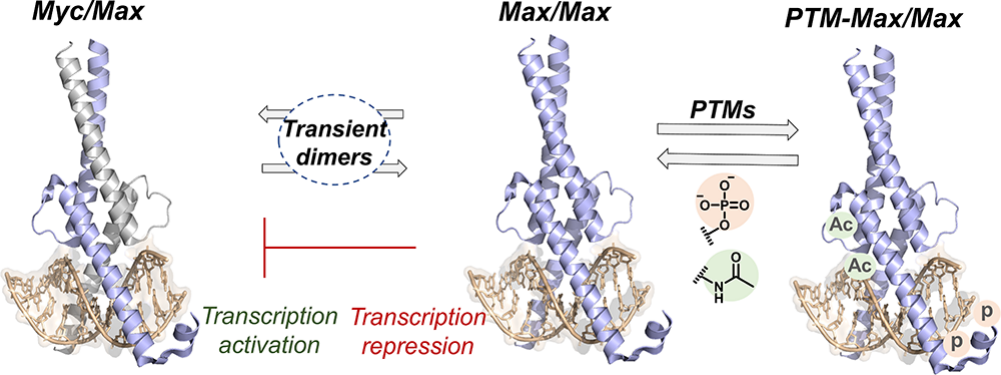
Post-translational modifications of the Max transcription factor modulate its function to either activate or repress gene expression; the acetylation and phosphorylation sites are highlighted in green and red, respectively (PDB code 1HLO).

Synthetic strategies to prepare complex modified proteins are essential in order to prepare novel proteins for numerous applications.^8,9^ The development of solid-phase peptide synthesis (SPPS)^10^ and chemoselective ligation technologies has significantly advanced protein science by enabling the efficient production of modified proteins with diverse transformations for numerous structural and functional studies.^11,12^ In this regard, native chemical ligation (NCL) has been widely utilized to produce proteins by combining peptides with an N-terminal Cys and a C-terminal thioester under aqueous conditions.^13^ The increasing demand to synthesize complex protein targets, particularly in multistep syntheses, has spurred the development of several innovative approaches to generate proteins with high homogeneity and at a workable scale.^14,15^ Importantly, the strategy and sequence in which peptide segments are reacted play a crucial role in determining the synthesis time,^16−18^ yield,^19−21^ and quality of the final product.^22−24^ These approaches include peptide ligation on solid support,^25^ convergent assembly,^26^ and one-pot synthesis.^27^ Such approaches enabled the effective production of novel modified proteins of varying sizes and complexities.^28,29^ Despite advances in chemical protein synthesis, the production of heteromodified TFs with defined modifications (e.g., phosphorylation and acetylation) remains scarce.^30^ Here, we report a practical one-pot synthesis of full-length Max TF analogues using NCL. This strategy enabled the isolation of homogeneous Max analogues, including site-specifically modified Max bearing both phosphorylated and acetylated residues on a milligram scale. Importantly, the reported synthesis enabled us to probe the effect of the crosstalk between Ser-phosphorylation and Lys-acetylation on Max function, which was found to significantly inhibit the DNA-binding activity.

Chemical protein synthesis offers a robust method for preparing complex proteins bearing desired transformations at selected sites. The flexibility of incorporating virtually any desired residue during SPPS, coupled with the power of chemoselective ligation technology, has enabled the production of proteins with various PTMs (e.g., phosphorylation, acetylation, methylation, and others).^31,32^ For example, we have recently reported the chemical synthesis of full-length Max TF bearing site-specific Lys-acetylation or Ser-phosphorylation marks via sequential NCL reactions.^6,7^ Interestingly, we found that the order in which the peptide segments are ligated (e.g., N-to-C terminus) significantly impacts both the synthesis yield and the quality of the final products. To further facilitate the production of Max analogues, we sought to develop a practical one-pot synthetic strategy to isolate homogeneously modified analogues bearing novel PTM patterns to probe the impact of the crosstalk of these natural transformations on the DNA-binding activity of Max TF.

To explore the effect of the crosstalk between Ser-phosphorylation and the Lys-acetylation marks on Max function, we decided to synthesize doubly acetylated and phosphorylated Max. To this end, we divided the sequence of Max into three segments, Cys-Max(93–151)-K segment **1**, Cys-Max(53–91)-NHNH_2_ segment **2**, Cys-MaxK57Ac(53–91)-NHNH_2_ segment **2′**, Max(1–51)-NHNH_2_ segment **3**, and MaxS2pS11pK31Ac(1–51)-NHNH_2_ segment **3′** (Figure 2). The N-terminal Ala residues in segments **2**, **2′**, and **1** were temporarily mutated to Cys to enable NCL at Ala-52 and Ala-92 junctions, and the thioester segments were prepared using the peptide-hydrazide approach.^33^ We prepared all peptide segments using the standard Fmoc-SPPS method (see section 4 of the Supporting Information). This design allowed for the isolation of all segments (**1**, **2**, **2′**, **3**, and **3′**) in yields of 11, 19, 21, 15, and 8%, respectively, after purification by preparative reversed-phase high-performance liquid chromatography (RP-HPLC).

**Figure 2:**
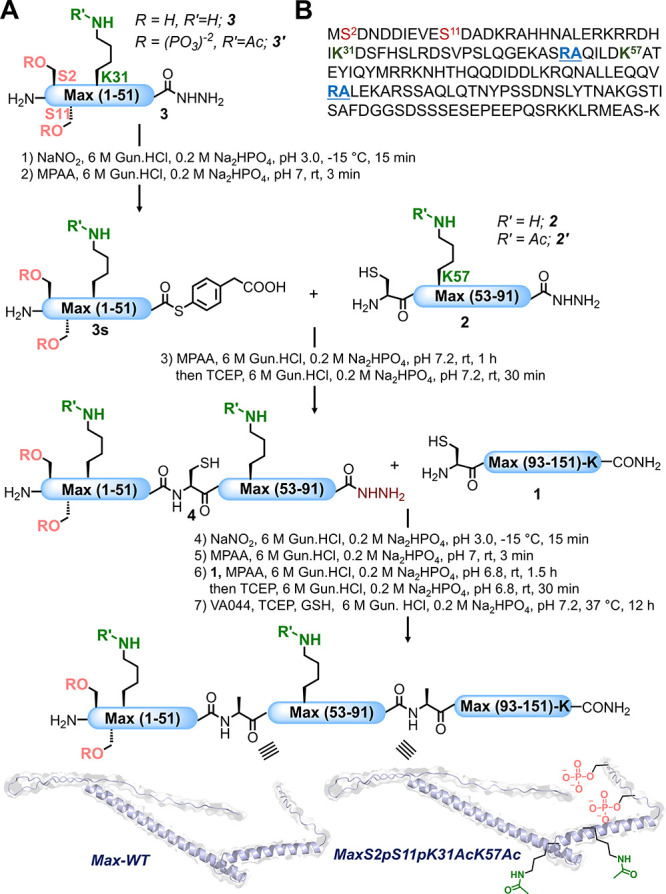
One-pot total chemical synthesis of a site-specifically modified Max transcription factor. (A) Max synthesis through one-pot native chemical ligation coupled with desulfurization. (B) Max sequence (p21 isoform); the ligation sites are highlighted in blue. The Met-1, Met-65, and Met-148 residues were substituted with the homologous norleucine (Nle) analogues to prevent oxidation.

After preparing all peptide segments, we initially set out to explore the potential of one-pot synthesis for the full-length native Max (**Max-WT**). We started the synthesis by converting segment **3**, Max(1–51)-NHNH_2_, to acyl-azide using NaNO_2_ in a 6 M Gun·HCl, 0.2 M Na_2_HPO_4_ buffer at pH 3.0 and −15 °C for 15 min. Subsequently, 4-mercaptophenylacetic acid (MPAA) was added in a 6 M Gun·HCl, 0.2 M Na_2_HPO_4_ buffer at pH 7 for *in situ* thioesterification to generate a **3s** intermediate (see the section 5 of the Supporting Information). Then, the thioester intermediate **3s** was ligated with Cys-Max(53–91)-NHNH_2_**2** and incubated for 1 h at room temperature. Afterward, the reducing reagent, tris(2-carboxyethyl)phosphine hydrochloride (TCEP·HCl), was added to the reaction mixture, and the reaction was kept for an additional 30 min (Figure 2A). The progress of the ligation was monitored by liquid chromatography–mass spectrometry (LC–MS) (Figure 3A), which confirmed the formation of the desired product **4** after 1.5 h. Before the following ligation, the reaction mixture was desalted, and the pH of the reaction was adjusted to ∼3 using 5 N HCl to convert intermediate **4** to a peptide thioester using NaNO_2_, followed by the addition of MPAA as described above. Then, peptide thioester **4s** was reacted with segment **1** to afford the full-length Max analogue after 2 h, as confirmed by LC–MS. Finally, the crude reaction mixture was desalted and subjected to desulfurization using 2,2′-azobis[2-(2-imidazolin-2-yl)propane] dihydrochloride (VA044), along with TCEP, and glutathione (GSH) to convert Cys-52 and Cys-92 to native Ala.^34,35^ The native Max (**Max-WT**) was obtained in 25% overall isolated yield after the seven synthetic steps, with a single RP-HPLC purification.

**Figure 3:**
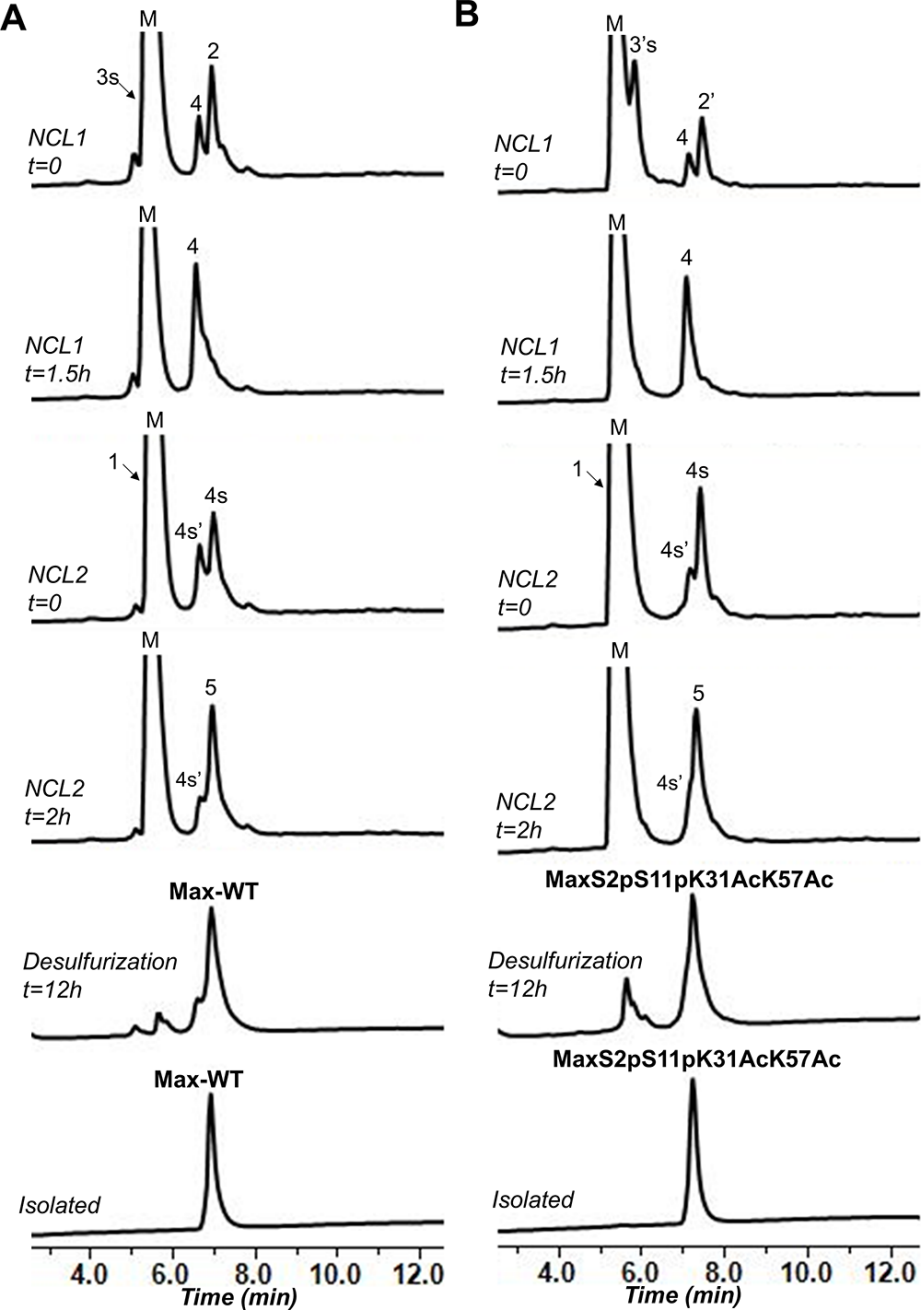
Analytical HPLC traces of the chemical synthesis and isolation of Max analogues. (A) One-pot NCL and desulfurization reactions to prepare **Max-WT**. (B) One-pot NCL and desulfurization reactions to prepare **MaxS2pS11pK31AcK57Ac**. 4s′, hydrolysis of 4s; M, MPAA.

We employed the same strategy described above to prepare a dual-modified Max bearing doubly phosphorylated and acetylated marks (**MaxS2pS11pK31AcK57Ac**). First, we converted segment **3′** (MaxS2pS11pK31Ac(1–51)-NHNH_2_) to peptide thioester and reacted it with segment **2′** Cys-MaxK57Ac(53–91)-NHNH_2_ using NaNO_2_, MPAA, and TCEP to afford the intermediate **4**, MaxS2pS11pK31AcK57Ac(1–91)-NHNH_2_, after 1.5 h (Figure 3B). Next, the reaction mixture was desalted and intermediate **4** was converted to peptide thioester and ligated with peptide segment **1** Cys-Max(93–151)-K, affording the target modified Max intermediate. Finally, the reaction mixture was desalted and underwent a one-pot desulfurization employing VA044, TCEP, and GSH to furnish the final product, **MaxS2pS11pK31AcK57Ac**, in 26% overall isolated yield. The identity and quality of the final isolated products, **Max-WT** and **MaxS2pS11pK31AcK57Ac**, were confirmed via LC–MS (Figure 4A). Importantly, the success in isolating full-length Max analogues in good yield and in a rapid manner (<20 h), starting from three peptide segments, highlights the power of our strategy to prepare libraries of modified Max analogues for various applications.

**Figure 4:**
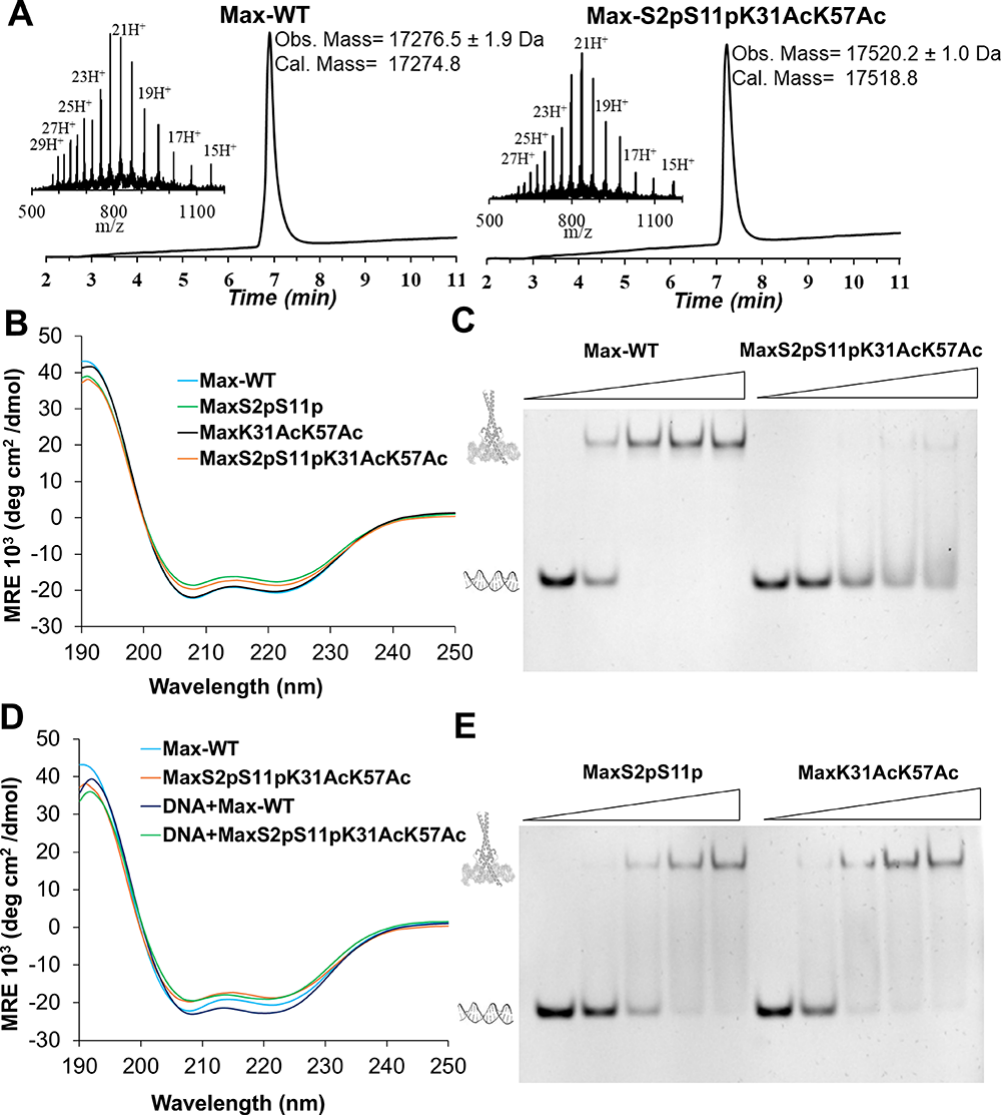
Max acetylation and phosphorylation significantly reduce its DNA-binding activity. (A) LC–MS analysis of isolated Max analogues. Analytical LC–MS of purified **Max-WT** with the observed mass 17276.5 ± 1.9 Da, the calculated mass 17274.8 Da (average isotopes), and purified **MaxS2pS11pK31AcK57Ac** with the observed mass 17520.2 ± 1.0 Da, and the calculated mass 17518.8 Da (average isotopes). The ultraviolet (UV) absorbance was monitored at 214 nm, and the *m*/*z* data were acquired over the entire peak. (B) CD analysis of synthetic Max analogues. (C) EMSA experiment of Max variants and the E-box DNA probe. (D) CD analysis of Max variants in the absence and presence of DNA. (E) EMSA experiment of individual phosphorylated and acetylated Max variants and the E-box DNA probe. EMSA conditions: 1 μM DNA probe and 0, 2, 4, 6, and 8 μM of Max incubated in 10 mM MES, 150 mM KCl, 1 mM MgCl_2_, and 10% glycerol buffer (pH 6.0). CD conditions: 10 μM Max in 10 mM MES, 150 mM KCl, 1 mM MgCl_2_, and 10% glycerol buffer (pH 6.0). All CD analyses were performed in triplicate, and EMSA experiments in duplicate.

To investigate the crosstalk between phosphorylation and acetylation on Max’s DNA-binding activity, we first characterized the folding of the synthesized variants **MaxS2pS11pK31AcK57Ac** and native **Max-WT**, as well as the distinct doubly acetylated and phosphorylated analogues **MaxK31AcK57Ac** and **MaxS2pS11p**, using circular dichroism (CD) spectroscopy (see the section 7 of the Supporting Information). In these experiments, all Max analogues exhibited the expected α-helical structures, with deep double minima at 208 and 222 nm, confirming proper folding into an α-helical structure (Figure 4B). Notably, although all four analogues adopted the expected α-helical folding, we observed a reduction in α-helicity in the **MaxS2pS11pK31AcK57Ac** and **MaxS2pS11p** analogues, as manifested by a decrease in the molar ellipticity curves. To probe this effect, we calculated the α-helical content of the four Max analogues using the Luo–Baldwin formula.^36^ We found that the phosphorylated analogues **MaxS2pS11p** and **MaxS2pS11pK31AcK57Ac** reduced the α-helical content of Max by approximately 5–8% compared with the native **Max-WT** (see section 7 of the Supporting Information). Importantly, as previously reported, the acetylation of Max (e.g., **MaxK31AcK57Ac**) did not interfere with Max’s folding (Figure 4B).^7^ These data indicate that Ser- phosphorylation can destabilize the secondary structure of Max and alter its folding.

The phosphorylation and acetylation modifications significantly reduce Max’s DNA-binding activity. Initially, we incubated both Max variants (**MaxS2pS11pK31AcK57Ac** and **Max-WT**) separately with the E-box DNA probe and evaluated the DNA-binding activity using an electrophoretic mobility shift assay (EMSA; see section 8 of the Supporting Information). Importantly, **Max-WT** associated with the DNA probe in a dose–response manner (Figure 4C). Remarkably, we observed substantial reduction in the DNA-binding activity of **MaxS2pS11pK31AcK57Ac**, with very minor binding at elevated concentrations. We confirmed via size-exclusion chromatography (SEC) that this inhibition is not due to the disruption of Max homodimerization (see the section 9 of the Supporting Information). These analyses revealed that both Max variants efficiently folded to form the 35 kDa homodimeric complex with comparable efficiency, confirming that phosphorylation and acetylation do not interfere with the Max dimerization but disrupt essential interactions with DNA.

We then assessed the reduction in DNA-binding activity by determining the dissociation constant (*K*_D_) of **Max-WT** and **MaxS2pS11pK31AcK57Ac** using biolayer interferometry (BLI; see the section 10 of the Supporting Information). We determined a *K*_D_ of 15.26 ± 0.33 nM for **Max-WT**. Importantly, we observed a ∼70-fold increase in the *K*_D_ of **MaxS2pS11pK31AcK57Ac** (*K*_D_ = 1.10 ± 0.03 μM). These findings further confirm the significant impact of phosphorylation and acetylation on inhibiting the DNA-binding activity of Max. This effect was further confirmed by performing CD analysis in the absence and presence of DNA (Figure 4D). Notably, we did not observe any structural stabilization of **MaxS2pS11pK31AcK57Ac** in the presence of DNA, due to the diminished DNA-binding. However, we observed a clear improvement in the helicity of **Max-WT** in the presence of DNA. These findings further confirm the functional DNA-binding activity of **Max-WT** and the reduced DNA-binding of **MaxS2pS11pK31AcK57Ac**. Remarkably, the DNA-binding inhibition of **MaxS2pS11pK31AcK57Ac** was more pronounced than that observed for the individual phosphorylated and acetylated analogues, **MaxS2pS11p** and **MaxK31AcK57Ac**, as confirmed by a side-by-side EMSA analysis (Figure 4E). These results indicate that the combined acetylation and phosphorylation PTMs markedly affect the activity of Max, compared with the individual phosphorylated or acetylated variants. This inhibition could be attributed to the destabilization of Max and the potential interference of these PTMs with essential DNA contacts and interactions. Although none of the acetylation (Lys-31/57) and phosphorylation (Ser-2/11) sites directly contact the E-box sequence, these residues are proximal to the DNA backbone.^37^ We anticipate that the significant inhibition in the DNA -binding of **MaxS2pS11pK31AcK57Ac**, compared with the other analogues, could result from a combination of disrupted electrostatic interactions between the Lys-31/Lys-57 and the DNA backbone as result of the acetylation, as well as electrostatic repulsion between the phosphorylated Ser-2/Ser-11 residues and the DNA backbone. Importantly, these experiments demonstrate that isolating homogeneous Max TF with site-specific phosphorylation and acetylation marks reveals that combining multiple PTMs significantly impacts protein function, as demonstrated by the significant reduction in the DNA-binding activity of Max.

We presented a rapid and efficient one-pot synthetic strategy to prepare the essential TF, Max, with site-specific phosphorylation and acetylation marks. By combining the peptide hydrazide approach with native chemical ligation and free-radical desulfurization, we successfully obtained Max analogues with high yields and excellent purity. Importantly, this approach enabled us to isolate dually modified Max TF, thus facilitating the exploration of the crosstalk between Ser- phosphorylation and Lys-acetylation in regulating the DNA-binding of Max. Through biochemical and biophysical analyses, we found that these post-translational modifications significantly impaired Max’s DNA-binding activity, most likely due to disruption of its folding and interaction with DNA. Our synthetic strategy provides a powerful tool for generating a variety of Max analogues with desired modifications at selected sites, providing insights into how the PTM mechanisms govern TF–DNA interactions and paving the way for better understanding and modulating gene expression programs.^38^

## Data Availability

The data underlying this study are available in the published article and its Supporting Information.

## Supporting Information Available

Synthesis and methods of all compounds, chemical synthesis of peptide segments, native chemical ligation and one-pot desulfurization, DNA-binding assessment and electrophoretic mobility shift assay, circular dichroism analysis, and biolayer interferometry binding assay (PDF)
