# Supplementary material for: One-Pot Total Synthesis of a Post-translationally Modified Max Transcription Factor Sheds Light on Ser-Phosphorylation and Lys-Acetylation Crosstalk in DNA Binding
Source: Org Lett. 2025 Mar 31;27(14):3760–4. doi: 10.1021/acs.orglett.5c00978 (PMC11998062; doi:10.1021/acs.orglett.5c00978)
Supplement: Supplementary file 1 — ol5c00978_si_001.pdf [file ol5c00978_si_001.pdf]

Supporting Information

One-Pot Total Synthesis of a Posttranslationally Modified Max Transcription Factor Sheds Light on Ser-Phosphorylation and Lys-Acetylation Crosstalk in DNA Binding

Raj V. Nithun, Shada Khoury, and Muhammad Jbara*

School of Chemistry, Raymond and Beverly Sackler Faculty of Exact Sciences, Tel Aviv University, Tel Aviv, 69978 Israel.

*Correspondence to: jbaram@tauex.tau.ac.il

Table of Contents

1. EXPERIMENTAL **3**

1.1 MATERIALS 3
1.2 ANALYTICAL LC-MS ANALYSIS 4
1.3 PREPARATIVE RP-HPLC PURIFICATION 4
1.4 pH MEASUREMENTS FOR LIGATION REACTIONS 4

2. PROTEIN AND DNA SEQUENCES **4**

3. PREPARATION OF 2-CHLOROTRITYL-HYDRAZINO-RESIN **5**

4. CHEMICAL SYNTHESIS OF THE PEPTIDE SEGMENTS **6**

4.1 SYNTHESIS OF SEGMENT 1 CYS-MAX(93-151)-K 6
4.2 SYNTHESIS OF SEGMENT 2 CYS-MAX(53-91)-$NHNH_2$ 8
4.3 SYNTHESIS OF SEGMENT 2' CYS-MAXK57AC(53-91)-$NHNH_2$ 9
4.4 SYNTHESIS OF SEGMENT 3 MAX(1-51)-$NHNH_2$ 11
4.5 SYNTHESIS OF SEGMENT 3' MAXS2PS11PK31AC(1-51)-$NHNH_2$ 13

5. CHEMICAL SYNTHESIS OF MAX VARIANTS VIA ONE-POT NCL-DESULFURIZATION **15**

5.1 ONE-POT CHEMICAL SYNTHESIS OF MAX-WT 15
5.2 ONE POT CHEMICAL SYNTHESIS OF MAXS2PS11PK31ACK57AC 18
5.3 CHEMICAL SYNTHESIS OF MAXS2PS11P AND MAXK31ACK57AC 21

6. FOLDING OF MAX VARIANTS **22**

7. CIRCULAR DICHROISM (CD) ANALYSIS **22**

8. DNA-BINDING ANALYSIS AND ELECTROPHORETIC MOBILITY-SHIFT ASSAY (EMSA) **24**

9. SIZE EXCLUSION CHROMATOGRAPHY (SEC) ANALYSIS **25**

10. OCTET BIOLAYER INTERFEROMETRY (BLI) BINDING ASSAY **25**

10.1 MAX-WT BLI BINDING ASSAY 25
10.2 MAXS2PS11PK31ACK57AC BLI BINDING ASSAY 26

11. REFERENCES **27**

1. Experimental

1.1 Materials

Fmoc-L-Phe-OH, Fmoc-L-Asn(Trt)-OH, Fmoc-L-Gln(Trt)-OH, Fmoc-L-Arg(Pbf)-OH, Fmoc-L-Tyr(tBu)-OH, Fmoc-L-Glu(OtBu)-OH, Fmoc-L-Val-OH, Fmoc-L-Ala-OH, Fmoc-L-Leu-OH, Fmoc-L-His(Trt)-OH, Fmoc-L-Asp(OtBu)-OH, Fmoc-L-Pro-OH, Fmoc-L-Cys(Trt)-OH, Fmoc-L-Lys(Boc)-OH, Fmoc-L-Lys(Alloc)-OH, Fmoc-L-Ile-OH, Fmoc-L-Thr(tBu)-OH, Fmoc-L-Ser(tBu)-OH, Fmoc-Gly-OH, Fmoc-L-Ser(PO(OBzl)OH)-OH, Fmoc-L-Nle-OH, Fmoc-L-Lys(Ac)-OH, Boc-L-Cys(Trt)-OH, Fmoc-L-Asp(OtBu)-(Dmb)Gly-OH, N,N´-Diisopropylcarbodiimide (DIC), Hydrazine hydrate-50% and Ethidium bromide were purchased from Sigma-Aldrich. Fmoc-L-Ser(ψMe,Mepro)-OH was purchased from Iris-Biotech. 1-[Bis(dimethylamino)methylene]-1H-1,2,3-triazolo[4,5-b]pyridinium 3-oxid hexafluorophosphate (HATU), (2-(1H-benzotriazol-1-yl)-1,1,3,3-tetramethyluronium hexafluorophosphate (HBTU), and 1-Hydroxybenzotriazole (HOBt) hydrate were purchased from Luxembourg Bio Technologies Ltd. Fmoc-L-His(Boc)-OH, HO-TCP(Cl)-ProTide resin and Rink Amide ProTide (LL) resin were obtained from CEM. Oligonucleotides were purchased from Integrated DNA Technologies (IDT, Coralville, IA). Diethyl ether (Et_2O, stabilized/BHT, AR grade), Dichloromethane (CH_2Cl_2, stabilized with amylene, peptide synthesis grade), N,N-dimethylformamide (DMF, peptide synthesis and AR grade), Acetonitrile (LC/MS and HPLC Grade), Trifluoroacetic acid (TFA, ≥99% ReagentPlus®), Diisopropylethylamine (DIEA, ≥99% ReagentPlus®), Piperidine (≥99% ReagentPlus®), Triisopropylsilane (TIS, 98%), Formic acid (98-100% for LC/MS), and Dimethyl sulfoxide (DMSO, ≥99.5% ReagentPlus®) were purchased from Bio-Lab ltd. TBE Running buffer (5X), Acrylamide Solution (40%), Tetramethylethylenediamine (TEMED), Ammonium Persulfate (APS), and 6X DNA Loading Dye were purchased from Thermo Fisher Scientific. Water for all reactions carried out on proteins and reverse-phase purification was obtained via deionized water filtration through a MilliporeSigmaTM Milli-QTM Ultrapure Water System. All chemicals obtained from the supplier were used as received without further purification.

1.2 Analytical LC-MS analysis

Analytical LC was acquired using Thermo Scientific Vanquish HPLC. The mobile phases used are solvent A (0.05% formic acid in water) and solvent B (0.05% formic acid in acetonitrile) and mass spectrometry using Thermo Scientific ISQ EM Mass spectrometer.

Method A: bioZen™ 2.6 µm-C4 Widepore LC column (150 x 2.1 mm); LC conditions: 5% B from 0–1.0 min, then a linear gradient from 5% to 50% B from 1.0–11.0 min (i.e., 4.5% per min), 0.3 mL/min flow rate.

1.3 Preparative RP-HPLC purification

Preparative RP-HPLC was performed using Thermo Scientific DIONEX UltiMate 3000 Variable Wavelength Detector, equipped with an XBridge® Protein BEH C4 OBD™ Prep column, 300 Å, 5 µm, (250 x 10 mm). Mobile phases used for LC analysis were solvent A (0.05% TFA in water), and solvent B (0.05% TFA in acetonitrile). The following LC methods were used:

Method A: 5% B from 0–5 min, then a linear gradient from 5% to 40% B from 5-40 min (i.e., 1% per min), 4 mL/min flow rate at 30 °C.

Method B: 5% B from 0–5 min, then a linear gradient from 5% to 20% B from 5-10 min, followed by another linear gradient from 20% to 60% B from 10-50 min (i.e. 1% per min), 4 mL/min flow rate at 30 °C.

1.4 pH measurements for ligation reactions

All pH values in aqueous 6 M Gun·HCl were determined using a VWR pH meter with a SENTEK electrode.

2. Protein and DNA sequences

Max(1-151):

M**S**^{2}DNDDIEVE**S**^{11}DADKRAHHNALERKRRDHI**K**^{31}DSFHSLRDSVPSLQGEKASRAQILD**K**^{57}ATEYIQYMRRKNHTHQ QDIDDLKRQNALLEQQVRALEKARSSAQLQTNYPSSDNSLYTNAKGSTISAFDGGSDSSSESEPEEPQSRKKLRMEAS-*K*

The Met residue at positions 1, 65, and 148 were replaced with the isologous norleucine (Nle) residue to avoid Met oxidation.

The Lys residue was added to the C-terminus of Max for late-stage insertion of reporter tag e.g., fluorescent molecule for our ongoing biochemical and biophysical studies.

E-box DNA probe:

5'-CCGGCTGA*CACGTG*GTATTAAT-3'

3. Preparation of 2-chlorotrityl-hydrazino-resin

The preparation was carried out by the following scheme:

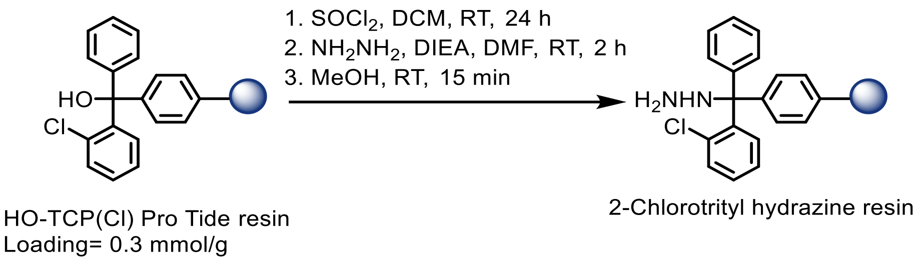

333.3 mg HO-TCP(Cl) resin (loading = 0.3 mmol/g, 0.1 mmol) was mixed with $SOCl_2$ (334 µL, 4.6 mmol, 1.5 M) and 2.7 ml of DCM in a septum-capped oven-dried round-bottom-flask that was cooled with N_2. The mixture was stirred overnight. $SOCl_2$ (134 µL, 1.8 mmol) was added the following morning and mixed for another 6 hours. The resin was then transferred into a fritted syringe and washed with DCM (8 mL X 6), DMF (8 mL X 3), and cooled to 0 ℃. A mixture of DIEA (190 µl, 1.1 mmol, 1.3 M) and hydrazine hydrate-50% (96 µl, 3.1 mmol, 3.6 M) in DMF (571 µl) was added slowly. Then, the suspension was stirred at room temperature. After 2 h, 115 µl MeOH was added to the reaction mixture to ensure the unreacted sites on the resin were capped and stirred for an additional 15 min. Finally, the resin was washed with DMF, H_2O, DMF, MeOH, and Et_2O and dried under a vacuum.[1]

4. Chemical synthesis of the peptide segments

4.1 Synthesis of segment 1 Cys-Max(93-151)-K

The synthesis was carried out according to the following scheme:

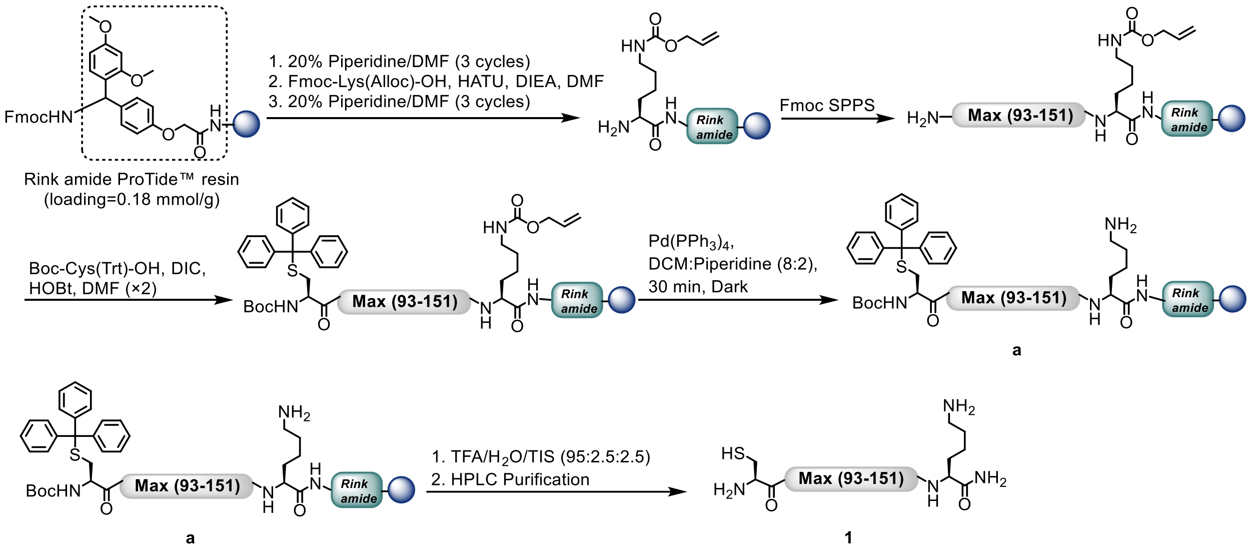

The synthesis of peptide segment **1** was carried out using stepwise Fmoc-SPPS on Rink amide ProTide™ resin (556 mg, loading 0.18 mmol/g, 0.1 mmol scale). The resin was pre-swollen in DMF for 30 min and then treated with 20% piperidine and 0.05% formic acid in DMF to remove the Fmoc protecting group. Subsequently, the functionalized resin was coupled with Fmoc-Lys(Alloc)-OH (4 equiv., 0.4 mmol, 0.19 M), using HATU (4 equiv., 0.4 mmol, 0.19 M) and 0.15 mL DIEA (0.86 mmol, 0.4 M) in 2 mL DMF for 2 h. The resin was then transferred to the CSBio automated peptide synthesizer and residues 151-128 were added in a stepwise fashion with Fmoc-amino acid (10 equiv., 1 mmol, 66.7 mM), using HBTU/HOBt (10 equiv., 1 mmol, 66.7 mM) and 0.37 mL DIEA (2 mmol, 133.3 mM) per amino acid. The coupling was carried out at room temperature (RT). Then the resin was taken out of the synthesizer and manually coupled with Fmoc-L-Asp(OtBu)-(Dmb)Gly-OH dipeptide (2.5 equiv., 0.25 mmol, 0.12 M) using HATU (2.5 equiv., 0.25 mmol, 0.12 M) and 0.08 mL DIEA (0.46 mmol, 0.22 M) in 2 mL DMF for 2 h. The resin was again transferred to the CSBio automated peptide synthesizer for continued stepwise addition of Fmoc-amino acids in a similar manner until amino acid residue 99. Residue 98 was coupled as Fmoc-L-Ser(ψMe,Mepro)-OH (5 equiv., 0.5 mmol, 0.23 M) using HBTU/HOBt (5 equiv., 0.5 mmol, 0.23 M) and 0.18 mL DIEA (1 mmol, 0.46 M). The remaining amino acids were coupled manually with Fmoc-amino acid (10 equiv., 1 mmol, 0.42 M), using HBTU/HOBt (10 equiv., 1 mmol, 0.42 M) and 0.37 mL DIEA (2 mmol, 0.84 M) in 2 mL DMF for 45 min. The last amino acid Boc-L-Cys(Trt)-OH (5 equiv., 0.5 mmol, 0.24 M) was coupled twice with HOBt/DIC

(5 equiv., 0.5 mmol, 0.24 M/5 equiv., 0.5 mmol, 0.24 M). Finally, to remove the Alloc protecting group, the resin was washed with DCM (5 mL x 3) and treated with $Pd(PPh_3)_4$ (1 equiv., 0.1 mmol) in DCM/piperidine (8/2, 4 ml) and shaken for 30 min at 25 °C under exclusion of light. The resin was then washed with DCM (5 mL x 3) and split. Half of the peptide resins were washed with DMF (5 mL x 3), MeOH (5 mL x 3), and DCM (5 mL x 3) and dried under a vacuum. To remove side chain protecting groups and release the peptide chains, a mixture of TFA/H_2O/TIS (95:2.5:2.5, 7 mL for 0.025 mmol scale) was added to each peptide resin and shaken for 3.5 h at RT. The resin was removed by filtration and washed with TFA (2 × 1 mL). To precipitate the peptide, the combined filtrate was added dropwise to cold diethyl ether (25 mL for 0.025 mmol resin) followed by centrifugation at 4000 rpm for 7 min. Then, the diethyl ether was decanted, followed by the dissolution of the peptide in 50% acetonitrile/water, diluted to 25% acetonitrile/water, and lyophilized to get crude segment **1** Cys-Max(93-151)-K (278 mg, 42.3 µmol) as a white powder. The crude dry peptide powder was purified by RP-HPLC (Method A described in Section 1.3), affording the product Cys-Max(93-151)-K (37 mg, 5.6 µmol, 11% yield based on 0.05 mmol resin) as a white powder.

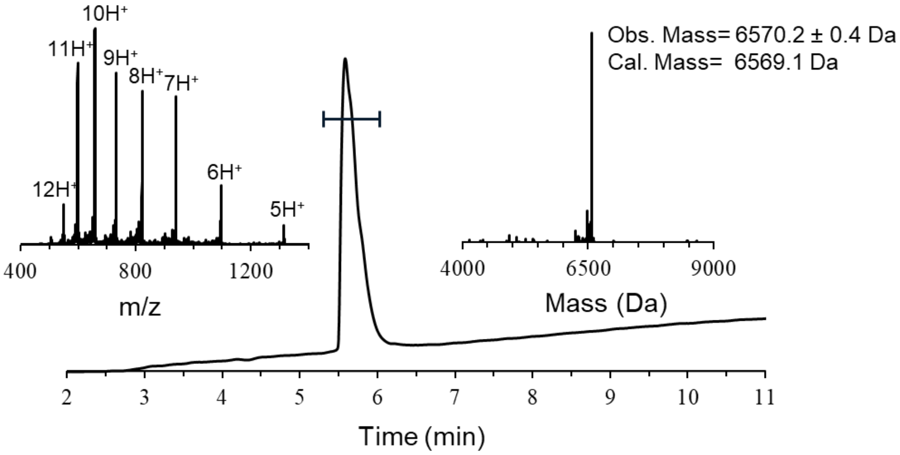

Figure S1. Analytical LC-MS analysis of segment **1** Cys-Max(93-151)-K with the observed mass 6570.2 ± 0.4 Da, calculated mass 6569.1 Da (average isotopes). The UV absorbance was monitored at 214 nm, and the mass-to-charge (m/z) data was acquired over the marked region in the chromatogram. LC-MS analysis was carried out with Method A depicted in section 1.2

4.2 Synthesis of segment 2 Cys-Max(53-91)-$NHNH_2$

The synthesis was carried out according to the following scheme:

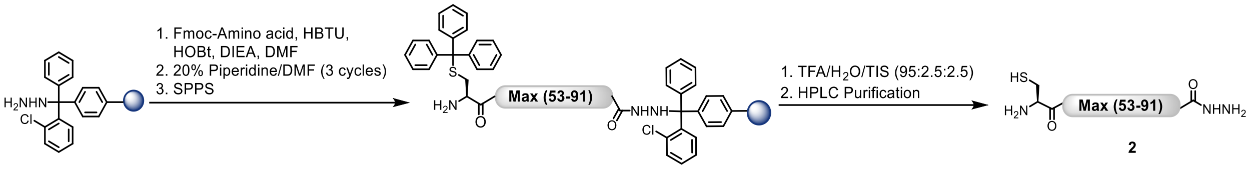

The synthesis of segment **2** Cys-Max(53-91)-$NHNH_2$ was carried out using stepwise Fmoc-SPPS on hydrazide resin (167 mg, loading 0.3 mmol/g, 0.05 mmol scale). The resin was pre-swollen in DMF for 30 min and then transferred to the CSBio automated peptide synthesizer and residues 91-52 added in a stepwise fashion with Fmoc-amino acid (10 equiv., 0.5 mmol, 33.3 mM), using HBTU/HOBt (10 equiv., 0.5 mmol, 33.3 mM) and 0.18 mL DIEA (20 equiv., 1 mmol, 66.7 mM). The coupling was carried out at 60 °C for 15 min of coupling time. When the synthesis was completed, the peptide resin was washed with DMF (5 mL x 3), MeOH (5 mL x 3), and DCM (5 ml x 3) and dried under a vacuum. To remove side chain protecting groups and release the peptide chains, a mixture of TFA/H_2O/TIS (95:2.5:2.5, 7 mL for 0.025 mmol scale) was added to the resin which was shaken for 3 h at RT. The resin was removed by filtration and washed with TFA (2 × 1 mL). To precipitate the peptide, the combined filtrate was added dropwise to cold diethyl ether (25 mL for 0.025 mmol resin) followed by centrifugation at 4000 rpm for 7 min. Then, the diethyl ether was decanted, followed by the dissolution of the peptide in 25% acetonitrile/water and lyophilized to get crude segment **2** Cys-Max(53-91)-$NHNH_2$ (216 mg, 43.7 µmol) as a white powder. The crude dry peptide powder was purified by RP-HPLC (Method B described in Section 1.3), affording the product Cys-Max(53-91)-$NHNH_2$ (46 mg, 9.3 µmol, 19% yield based on 0.05 mmol resin) as a white powder.

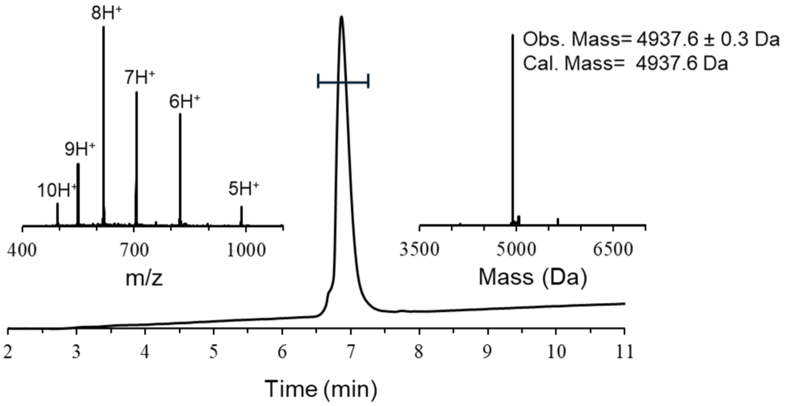

Figure S2. Analytical LC-MS analysis of segment **2** Cys-Max(53-91)-$NHNH_2$ with the observed mass 4937.6 ± 0.3 Da, calculated mass 4937.6 Da (average isotopes). The UV absorbance was monitored at 214 nm, and the m/z data was acquired over the marked region in the chromatogram. LC-MS analysis was carried out with Method A depicted in section 1.2.

4.3 Synthesis of segment 2’ Cys-MaxK57Ac(53-91)-$NHNH_2$

The synthesis was carried out according to the following scheme:

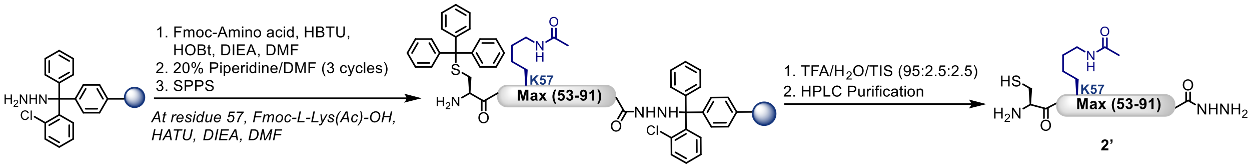

The synthesis of segment **2’** Cys-MaxK57Ac(53-91)-$NHNH_2$ was carried out using stepwise Fmoc-SPPS on hydrazide resin (167 mg, loading 0.3 mmol/g, 0.05 mmol scale). The resin was pre-swollen in DMF for 30 min and then transferred to the CSBio automated peptide synthesizer and residues 91-58 added in a stepwise fashion with Fmoc-amino acid (10 equiv., 0.5 mmol, 33.3 mM), using HBTU/HOBt (10 equiv., 0.5 mmol, 33.3 mM) and 0.18 mL DIEA (20 equiv., 1 mmol, 66.7 mM). The coupling was carried out at 30 °C for a 45 min coupling time. The resin was then taken out of the synthesizer and manually coupled with Fmoc-Lys(Ac)-OH (5 equiv., 0.25 mmol, 0.12 M) using HATU (5 equiv., 0.25 mmol, 0.12 M) and 90 µl DIEA (10 equiv., 0.5 mmol, 0.24 M) in 2 mL DMF for 1 h. After, the resin was transferred back to the CSBio automated peptide synthesizer and continued stepwise addition of the remaining amino acids in a similar manner. When the synthesis was completed, the peptide resin was washed with DMF (5 mL x 3), MeOH (5 mL x 3), and DCM (5 ml x 3) and dried under vacuum. To remove side chain protecting groups and release the peptide chains, a mixture of TFA/H_2O/TIS (95:2.5:2.5, 7 mL for 0.025 mmol scale) was added to the resin which was shaken for 3 h at RT. The resin was removed by filtration and washed with TFA (2 × 1 mL). To precipitate the peptide, the combined filtrate was added dropwise to cold diethyl ether (25 mL for 0.025 mmol resin) followed by centrifugation at 4000 rpm for 7 min. Then, the diethyl ether was decanted, followed by the dissolution of the peptide in 25% acetonitrile/water and lyophilized to get crude Cys-MaxK57Ac(53-91)-$NHNH_2$ (240 mg, 48.2 µmol) as a white powder. The crude dry peptide powder was purified by RP-HPLC (Method B described in Section 1.3), affording the product Cys-MaxK57Ac(53-91)-$NHNH_2$ (52 mg, 10.4 µmol, 21% yield based on 0.05 mmol resin) as a white powder.

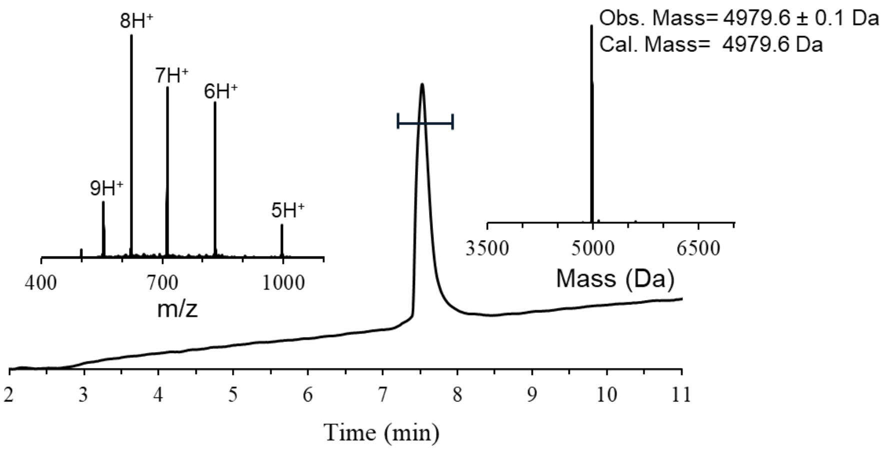

Figure S3. Analytical LC-MS analysis of segment **2'** Cys-MaxK57Ac(53-91)-$NHNH_2$ with the observed mass 4979.6 ± 0.1 Da, calculated mass 4979.6 Da (average isotopes). The UV absorbance was monitored at 214 nm and the m/z data was acquired over the marked region in the chromatogram. LC-MS analysis was carried out with Method A depicted in section 1.2.

4.4 Synthesis of segment 3 Max(1-51)-$NHNH_2$

The synthesis was carried out according to the following scheme:

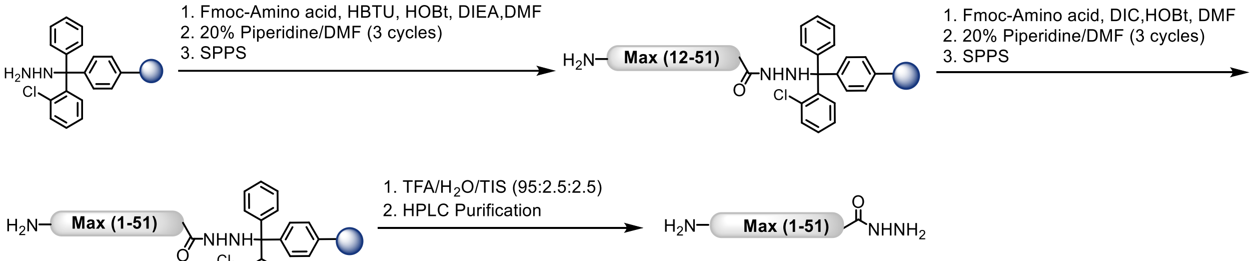

The synthesis of segment **3** Max(1-51)-$NHNH_2$ was carried out using stepwise Fmoc-SPPS on hydrazide resin (167 mg, loading 0.3 mmol/g, 0.05 mmol scale). The resin was pre-swollen in DMF for 30 min and then transferred to the CSBio automated peptide synthesizer and residues 51-12 added in a stepwise fashion with Fmoc-amino acid (10 equiv., 0.5 mmol, 33.3 mM), using HBTU/HOBt (10 equiv., 0.5 mmol, 33.3 mM) and 0.18 mL DIEA (20 equiv., 1 mmol, 66.7 mM). Residues 11-1 were then added with Fmoc-amino acid (10 equiv., 0.5 mmol, 33.3 mM) using DIC (10 equiv., 0.5 mmol, 33.3 mM) and HOBt (10 equiv., 0.5 mmol, 33.3 mM) in the same manner. All couplings were carried out at 30 °C for 45 min of coupling time. Finally, the peptide resin was washed with DMF (5 mL x 3), MeOH (5 mL x 3), and DCM (5 ml x 3) and dried under vacuum. To remove side chain protecting groups and release the peptide, a mixture of TFA/H_2O/TIS (95:2.5:2.5, 7 mL for 0.025 mmol scale) was added to the resin which was shaken for 3.5 h at RT. The resin was removed by filtration and washed with TFA (2 × 1 mL). To precipitate the peptide, the combined filtrate was added dropwise to cold diethyl ether (25 mL for 0.025 mmol resin) followed by centrifugation at 4000 rpm for 7 min. Then, the diethyl ether was decanted, followed by the dissolution of the peptide in 25% acetonitrile/water and lyophilized to get crude Max(1-51)-$NHNH_2$ (245 mg, 41.6 µmol) as a white powder. The crude dry peptide powder was purified by RP-HPLC (Method A described in Section 1.3), affording the product Max(1-51)-$NHNH_2$ (44 mg, 7.5 µmol, 15% yield based on 0.05 mmol resin) as a white powder.

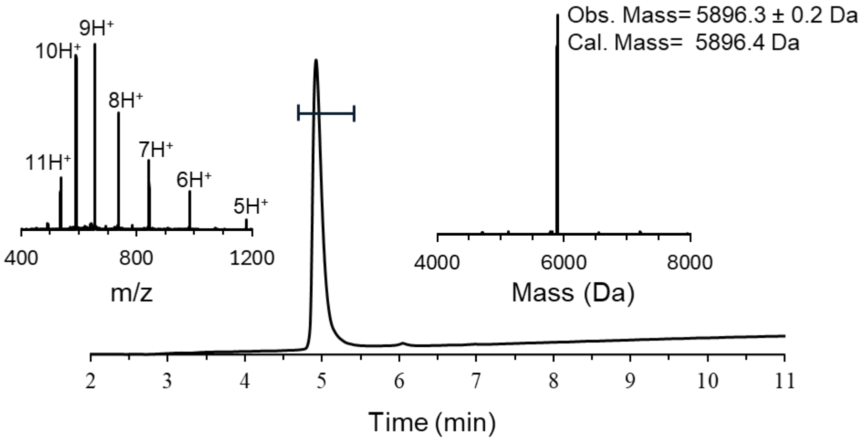

Figure S4. Analytical LC-MS analysis of segment **3** Max(1-51)-$NHNH_2$ with the observed mass 5896.3 ± 0.2 Da, calculated mass 5896.4 Da (average isotopes). The UV absorbance was monitored at 214 nm and the m/z data was acquired over the marked region in the chromatogram. LC-MS analysis was carried out with Method A depicted in section 1.2.

4.5 Synthesis of segment 3' MaxS2pS11pK31Ac(1-51)-$NHNH_2$

The synthesis was carried out according to the following scheme:

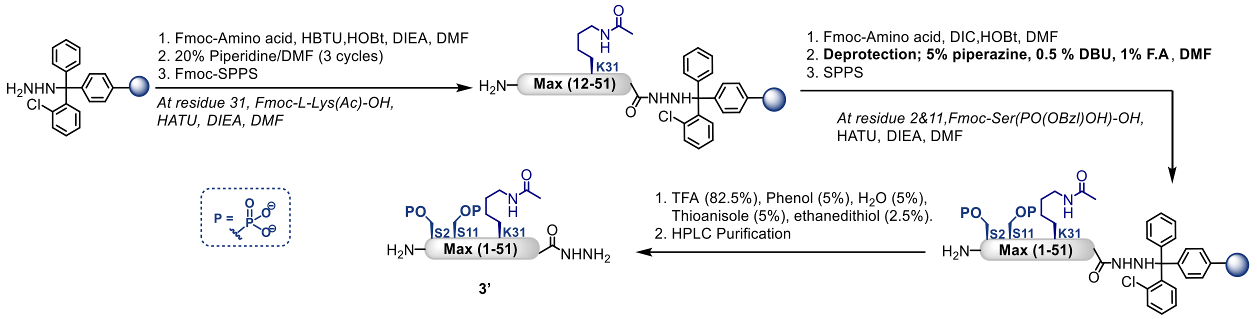

The synthesis of segment **3'** MaxS2pS11pK31Ac(1-51)-$NHNH_2$ was carried out using stepwise Fmoc-SPPS on hydrazide resin (167 mg, loading 0.3 mmol/g, 0.05 mmol scale). The resin was pre-swollen in DMF for 30 min and then coupled residues 51-32 in a stepwise fashion with Fmoc-amino acid (5 equiv., 0.25 mmol, 125 mM), using HBTU/HOBt (5 equiv., 0.25 mmol, 125 mM) and 90 µL DIEA (0.5 mmol, 250 mM). The coupling was carried out manually at 30 °C for 30 min coupling time. Then, coupled with Fmoc-Lys(Ac)-OH (5 equiv., 0.25 mmol, 125 mM) using HATU (5 equiv., 0.25 mmol, 125 mM) and 90 µL DIEA (10 equiv., 0.5 mmol, 250 mM) in 2 mL DMF for 1 h. After the resin was transferred to the CSBio automated peptide synthesizer and residues 30-12 were added in a stepwise fashion with Fmoc-amino acid (10 equiv., 0.5 mmol, 33.3 mM), using HBTU/HOBt (10 equiv., 1 mmol, 33.3 mM) and 0.18 mL DIEA (1 mmol, 66.7 mM). The coupling was carried out at 30 °C for 45 min coupling time. Following completing the synthesis until the 11th residue, the resin was taken out of the synthesizer and coupled using Fmoc-L-Ser(PO(OBzl)OH)-OH (2 equiv., 0.1 mmol, 50 mM) with HATU (2 equiv., 0.1 mmol, 50 mM) and DIEA (4 equiv., 0.2 mmol, 100 mM) for 2 h at RT. The Fmoc protecting group was then removed using 5% piperazine, 0.5 % DBU, and 1% FA in DMF.[2] The synthesis was continued manually using Fmoc-amino acid (10 equiv. 0.5 mmol, 250 mM), HOBt (10 equiv., 0.5 mmol, 250 mM), and DIC (10 equiv., 0.5 mmol, 250 mM). Fmoc deprotection was carried out using 5% piperazine, 0.5 % DBU, 1% FA in DMF. At residue 2, Fmoc-L-Ser(PO(OBzl)OH)-OH (2 equiv., 0.1 mmol, 50 mM) coupled with HATU (2 equiv., 0.1 mmol, 50 mM) and DIEA (4 equiv., 0.2 mmol, 100 mM) for 2 h at RT. Finally, residue 1 was coupled with Fmoc-Nle-OH (10 equiv. 0.5 mmol, 250 mM), HOBt (10 equiv., 0.5 mmol, 250 mM), and DIC (10 equiv., 0.5 mmol, 250 mM) and removed the Fmoc protecting group with 5% piperazine, 0.5 % DBU, and 1% F.A in DMF. The peptide-bound resin was then washed with DMF (5 mL x 3), MeOH (5 mL x 3), and DCM (5 ml x 3) and dried under a vacuum. Subsequently, a mixture of TFA (82.5%), Phenol (5%), Thioanisole (5%), H_2O (5%), and 1,2-ethanedithiol (2.5%) (7 mL for 0.025 mmol scale) was added to the resin, which was shaken for 3.5 h at

room temperature. The resin was removed by filtration and washed with TFA (2 × 1 mL). To precipitate the peptide, the combined filtrate was added dropwise to cold diethyl ether (25 mL for 0.025 mmol resin) followed by centrifugation at 4000 rpm for 7 min. Then, the diethyl ether was decanted, followed by the dissolution of the peptide in 25% acetonitrile/water and lyophilized to get crude MaxS2pS11pK31Ac(1-51)-$NHNH_2$ (170 mg, 27.9 µmol) as a white powder. The crude dry peptide powder was purified by RP-HPLC (Method A described in Section 1.3), affording the product MaxS2pS11pK31Ac(1-51)-$NHNH_2$ (24 mg, 3.9 µmol, 8 % yield based on 0.05 mmol resin) as a white powder.

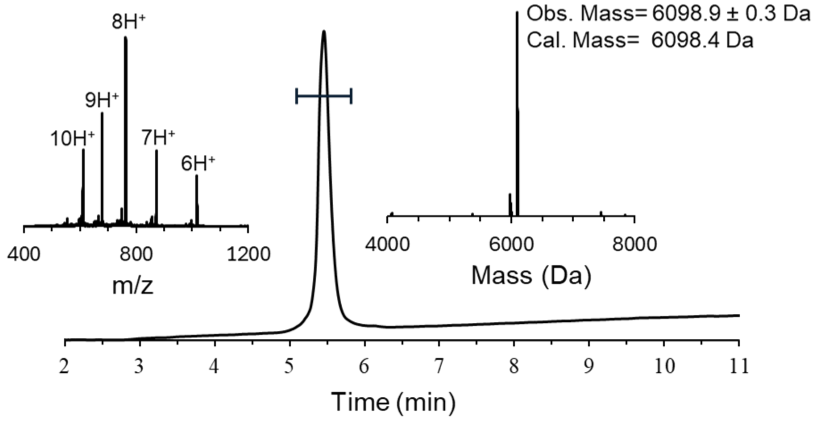

Figure S5. Analytical LC-MS analysis of segment **3'** MaxS2pS11pK31Ac(1-51)-$NHNH_2$ with the observed mass 6098.9 ± 0.3 Da, calculated mass 6098.4 Da (average isotopes). The UV absorbance was monitored at 214 nm and the m/z data was acquired over the marked region in the chromatogram. LC-MS analysis was carried out with Method A depicted in section 1.2.

5. Chemical synthesis of Max variants via one-pot NCL-desulfurization

5.1 One-pot chemical synthesis of Max-WT

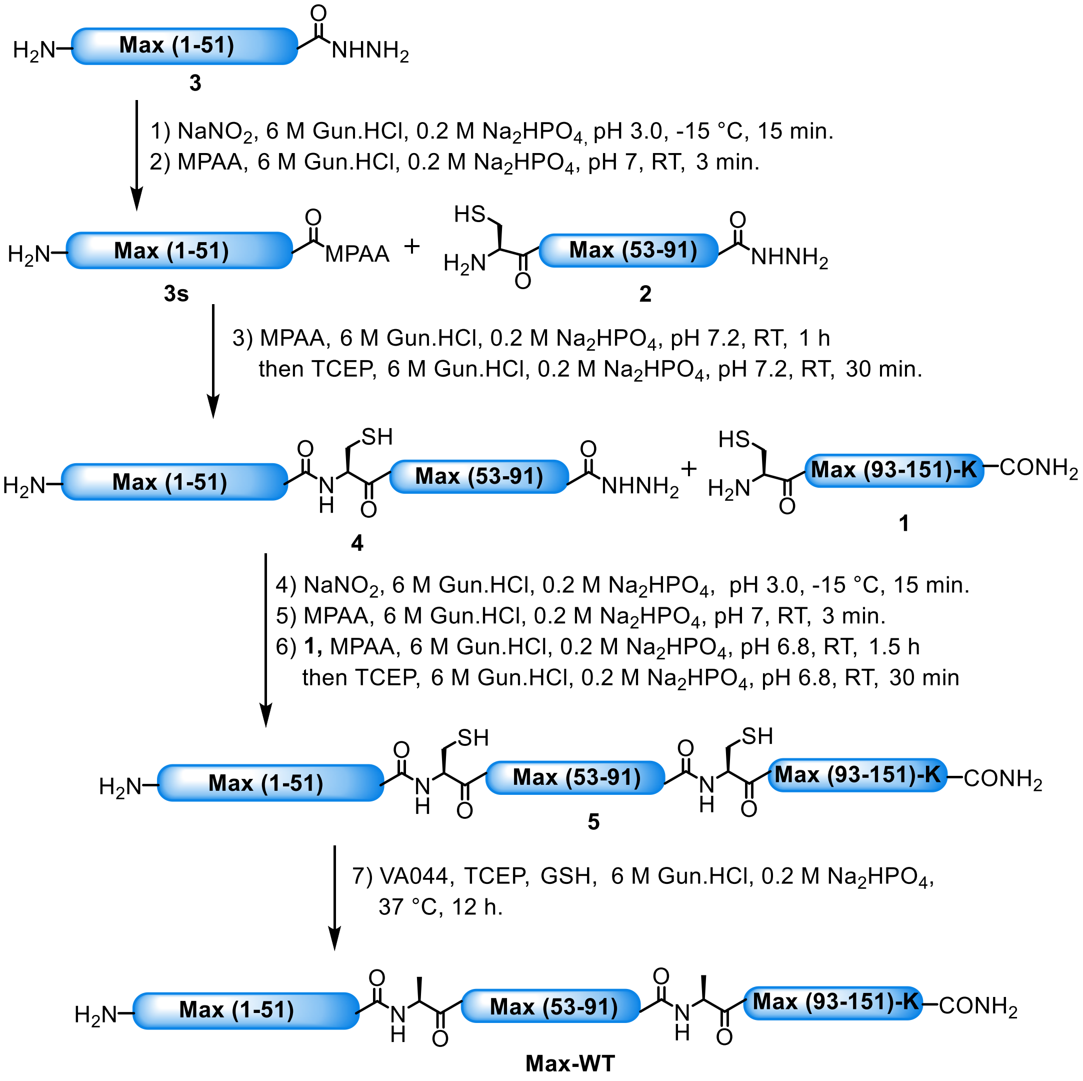

Segment **3** Max(1-51)-NHNH$_2$ (4.7 mg, 1.3 equiv., 0.8 µmol, 10.7 mM) was dissolved in 6 M Gun.HCl, 0.2 M Na_2HPO_4 buffer (75 µL) at pH 3.0 and cooled to -15 °C by placing in an ice/salt bath. 5 µL of $NaNO_2$ (10 equiv., 8.0 µmol, 1.6 M; based on **3**) dissolved in water was added to the reaction mixture and allowed to react for 15 min at -15 °C with gentle mixing in repeated intervals. After 15 min, 75 µL of MPAA (30 equiv., 24 µmol, 0.3 M; based on **3**) in 6 M Gun.HCl, 0.2 M Na_2HPO_4 buffer at pH 7 was added to the mixture and gently mixed for two to three min. Then, segment **2** Cys-Max(53-91)-NHNH$_2$ (3 mg, 1.0 equiv., 0.6 µmol, 4 mM) was dissolved in the reaction mixture and the pH was adjusted to 7.2 using 5 *N* NaOH. The mixture was then incubated for 1 h at 25 °C, and then 75 µL of TCEP (20 equiv., 16 µmol, 0.2 M; based on **3**) in 6 M Gun.HCl, 0.2 M Na_2HPO_4 buffer at pH 7.2 was added and continued incubating for 30 min at 25 °C. The

reaction was monitored using analytical LC-MS (Method A described in Section 1.2).[3,4] After 1.5 h ligation, the reaction mixture was desalted by pipetting the reaction mixture into a 10 kDa molecular weight cutoff spin filter (Amicon® Ultra- 2mL, 10K). The reaction mixture was diluted with a 3 M Gun.HCl, 0.1 M Na_2HPO_4 buffer (pH 7.2) to 2.0 mL and concentrated to 0.5 mL by Centrifuging the spin filter at 5000 rpm for 15 min. This process was repeated two times. Then the mixture was diluted with a 6 M Gun.HCl, 0.2 M Na_2HPO_4 buffer (pH 7.2) to 2.0 mL and concentrated to 200 µL, the reaction mixture was collected by reverse centrifuge and adjusted the pH to 3 using 5 *N* HCl. The reaction mixture was then cooled to -15 °C by placing it in an ice/salt bath. 5 µL of $NaNO_2$ (15 equiv., 9.2 µmol, 1.8 M; based on **2**) was dissolved in water and added to the reaction mixture and allowed to react for 15 min at -15 °C with gentle mixing in repeated intervals. After 15 min, 100 µL of MPAA (50 equiv., 30.5 µmol, 0.3 M; based on **2**) in 6 M Gun.HCl, 0.2 M Na_2HPO_4 buffer at pH 7 was added to the mixture and gently mixed for two to three min. The segment **1** Cys-Max(93-151)-K (4 mg, 1 equiv., 0.6 µmol, 2 mM; based on **2**) was then dissolved in the reaction mixture and the pH was adjusted to 6.8 using 5 *N* NaOH. The mixture was then incubated for 1.5 h at 25 °C and then 100 µL of TCEP (40 equiv., 24.4 µmol, 0.2 M; based on **2**) in 6 M Gun.HCl, 0.2 M Na_2HPO_4 buffer at pH 6.8 was added and continued incubating for 30 min at 25 °C. The reaction was monitored using analytical LC-MS (Method A described in Section 1.2). The ligation was completed in 2 h. After completion of the reaction, the crude reaction was desalted by pipetting the reaction mixture into a 10 kDa molecular weight cutoff spin filter (Amicon® Ultra- 2mL, 10K). The reaction mixture was diluted with a 3 M Gun.HCl, 0.1 M Na_2HPO_4 buffer (pH 7.2) to 2.0 mL and concentrated to 0.5 mL by centrifuging the spin filter at 5000 rpm for 15 min. This process was repeated two times more. Then, the mixture was diluted with a 6 M Gun.HCl, 0.2 M Na_2HPO_4 buffer (pH 7.2) to 2.0 mL and concentrated to 0.5 mL, the reaction mixture was collected by reverse centrifuge and then treated with VA044 (0.1 mmol, 200 mM), TCEP (0.13 mmol, 250 mM), and L-Glutathione (GSH, 30 µmol, 60 mM) for 12 h.[5,6] The progress of the reaction was monitored by analytical LC-MS using Method A (Section 1.2). After the completion of the reaction, purification was carried out using RP-HPLC (Method B described in Section 1.3) affording 2.6 mg (0.15 µmol) of the final product **Max-WT** as a white powder (25% yield, based on the limiting segment **2**).

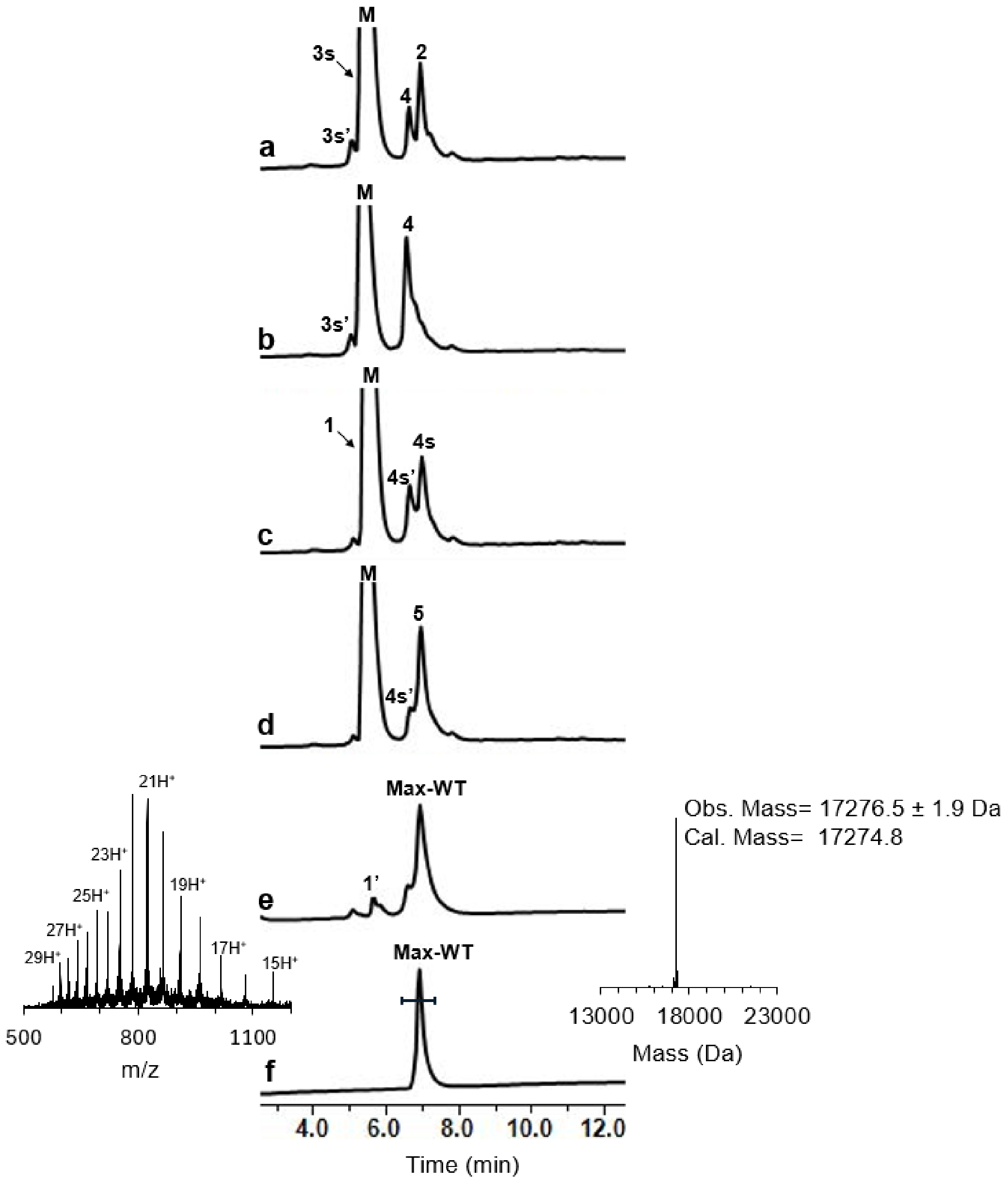

Figure S6. Analytical LC-MS of the progress of the one-pot synthesis of **Max-WT**. **(a)** first ligation at t = 0 min; Max(1-51) thioester segment (**3s**), hydrolysis of Max(1-51) thioester (**3s'**), Cys-Max(53-91)-$NHNH_2$ segment (**2**), ligated product Max(1-91)-$NHNH_2$ (**4**), and M=MPAA. **(b)** Crude first ligation reaction at t = 90 min. **(c)** Second ligation at t = 0 min; Max(1-91) thioester segment (**4s**), hydrolysis of Max(1-91) thioester (**4s'**), and Cys-Max(93-151)-K segment (**1**). **(d)** Crude ligation reaction at t = 120 min; ligated product (**5**) **(e)** Crude desulfurization reaction at t = 12 h; desulfurized segment **1** (**1'**). **(f)** RP-HPLC purified **Max-WT** with the observed mass 17276.5 ± 1.9 Da, calculated mass 17274.8 Da (average isotopes). The UV absorbance was monitored at 214 nm and the m/z data was acquired over the marked region in the chromatogram.

5.2 One pot chemical synthesis of MaxS2pS11pK31AcK57Ac

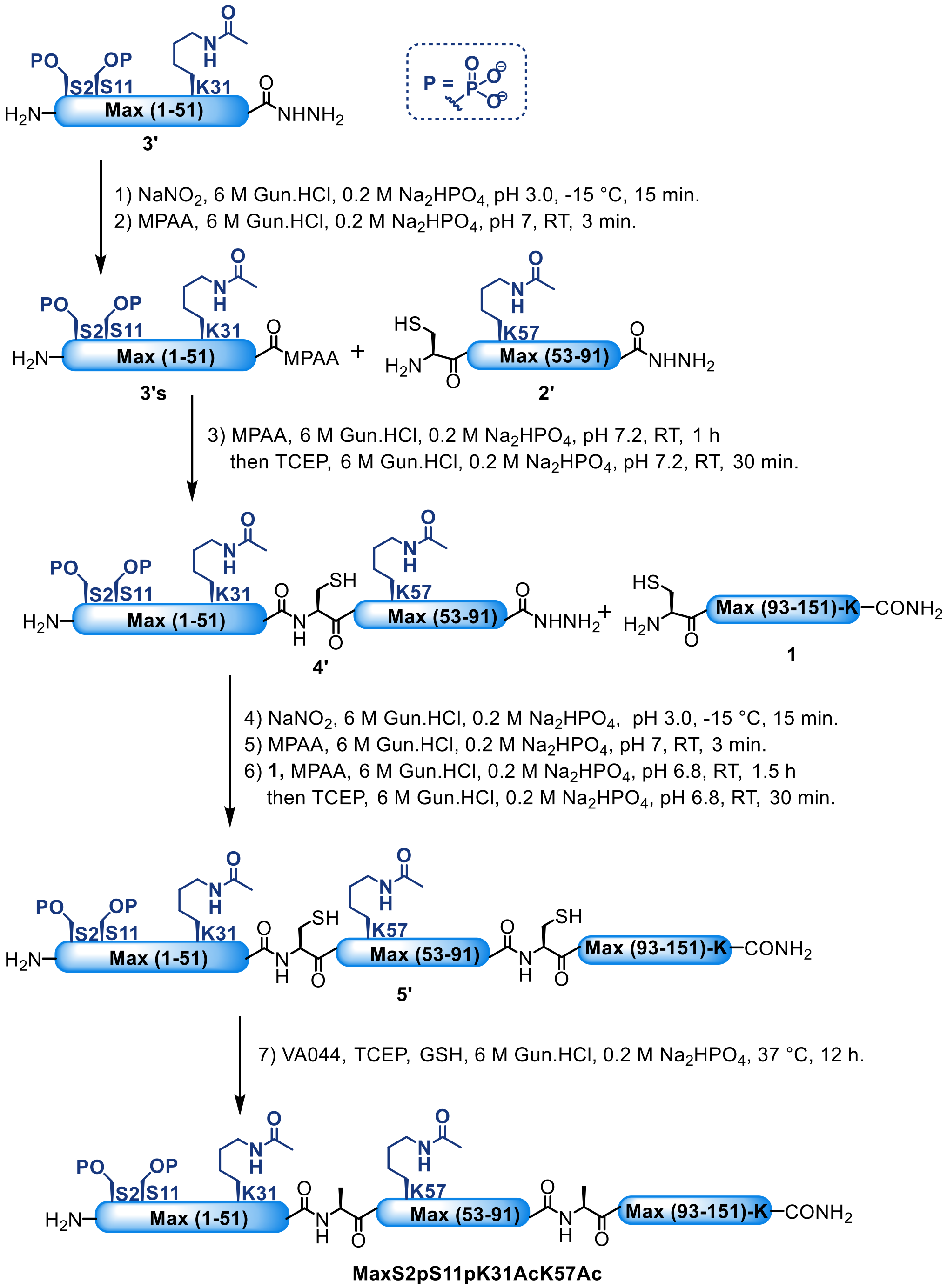

Segment **3’** MaxS2pS11pK31Ac(1-51)-$NHNH_2$ (3.2 mg, 1.3 equiv., 0.5 µmol, 10.4 mM) was dissolved in 6 M Gun.HCl, 0.2 M Na_2HPO_4 buffer (50 µL) at pH 3.0 and cooled to -15 °C by placing in an ice/salt bath. 5 µL of $NaNO_2$ (10 equiv., 5.2 µmol, 1.0 M; based on **3’**), which was dissolved in water, was added to the reaction mixture and allowed to react for 15 min at -15 °C with gentle mixing in repeated intervals. After

15 min, 50 µL of MPAA (30 equiv., 15.6 µmol, 0.3 M; based on **3'**) in 6 M Gun.HCl, 0.2 M Na_2HPO_4 buffer at pH 7 was added to the mixture and gently mixed for two to three min. Then, segment **2'** Cys-MaxK57Ac(53-91)-$NHNH_2$ (2 mg, 1.0 equiv., 0.4 µmol, 4 mM) was dissolved in the reaction mixture and the pH was adjusted to 7.2 using 5 *N* NaOH. The mixture was then incubated for 1 h at 25 °C and then 50 µL of TCEP (20 equiv., 10.4 µmol, 0.2 M; based on **3'**) in 6 M Gun.HCl, 0.2 M Na_2HPO_4 buffer at pH 7.2 was added and continued incubating for 30 min at 25 °C. The reaction was monitored using analytical LC-MS (Method A described in Section 1.2). After 1.5 h ligation, the reaction mixture was desalted by pipetting the reaction mixture into a 10 kDa molecular weight cutoff spin filter (Amicon® Ultra- 2mL, 10K). The reaction mixture was diluted with a 3 M Gun.HCl, 0.1 M Na_2HPO_4 buffer (pH 7.2) to 2.0 mL and concentrated to 0.5 mL by centrifuging the spin filter at 5000 rpm for 15 min. This process was repeated two times. Then, the mixture was diluted with a 6 M Gun.HCl, 0.2 M Na_2HPO_4 buffer (pH 7.2) to 2.0 mL and concentrated to 200 µL, the reaction mixture was collected by reverse centrifuge and the pH was adjusted to 3 using 5 *N* HCl. The reaction mixture was then cooled to -15 °C by placing it in an ice/salt bath. 5 µL of $NaNO_2$ (15 equiv., 6 µmol, 1.2 M; based on **2'**), which was dissolved in water, was added to the reaction mixture and allowed to react for 15 min at -15 °C with gentle mixing in repeated intervals. After 15 min, 50 µL of MPAA (50 equiv., 20 µmol, 0.4 M; based on **2'**) in 6 M Gun.HCl, 0.2 M Na_2HPO_4 buffer at pH 7 was added to the mixture and gently mixed for two to three min. The segment **1** Cys-Max(93-151)-K (2.6 mg, 1 equiv., 0.40 µmol, 1.6 mM; based on **2'**) was then dissolved in the reaction mixture and the pH was adjusted to 6.8 using 5 *N* NaOH. The mixture was then incubated for 1.5 h at 25 °C and then 50 µL of TCEP (40 equiv., 16 µmol, 0.3 M; based on **2'**) in 6 M Gun.HCl, 0.2 M Na_2HPO_4 buffer at pH 6.8 was added and continued incubating for 30 min at 25 °C. The reaction was monitored using analytical LC-MS (Method A described in Section 1.2). The ligation was completed in 2 h. After completion of the reaction, the crude reaction was desalted by pipetting the reaction mixture into a 10 kDa molecular weight cutoff spin filter (Amicon® Ultra- 2mL, 10K). The reaction mixture was diluted with a 3 M Gun.HCl, 0.1 M Na_2HPO_4 buffer (pH 7.2) to 2.0 mL and concentrated to 0.5 mL by centrifuging the spin filter at 5000 rpm for 15 min. This process was repeated two times more. Then, the mixture was diluted with a 6 M Gun.HCl, 0.2 M Na_2HPO_4 buffer (pH 7.2) to 2.0 mL and concentrated to 0.5 mL, the reaction mixture was collected by reverse centrifuge and then treated with VA044 (0.1 mmol, 200 mM), TCEP (0.13 mmol, 250 mM), and L-Glutathione (GSH, 30 µmol, 60 mM) for 12 h. The progress of the reaction was monitored by analytical LC-MS using Method A (Section 1.2). After the completion of the reaction, purification was carried out using RP-HPLC (Method B described in Section 1.3), affording 1.8 mg (0.10 µmol) of the final product **MaxS2pS11pK31AcK57Ac** as a white powder (26% yield, based on the limiting segment **2'**).

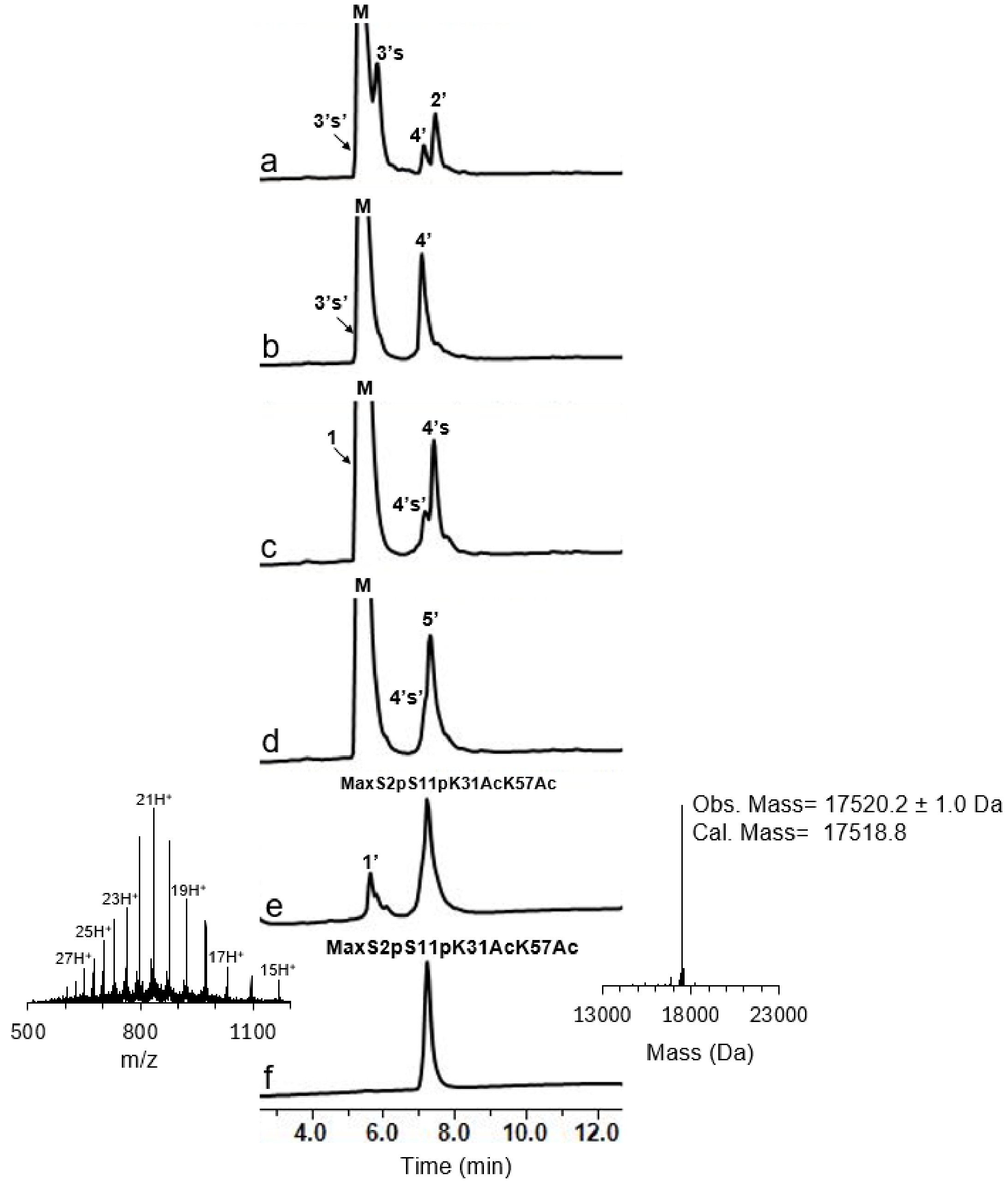

Figure S7. Analytical LC-MS of the progress of the one-pot synthesis of **MaxS2pS11pK31AcK57Ac**. **(a)** first ligation at t = 0 min; MaxS2pS11pK31Ac(1-51) thioester segment (**3's**), hydrolysis of MaxS2pS11pK31Ac(1-51) thioester (**3's'**), Cys-MaxK57Ac(53-91)-$NHNH_2$ segment (**2'**), ligated product MaxS2pS11pK31AcK57Ac(1-91)-$NHNH_2$ (**4'**), and M=MPAA. **(b)** Crude first ligation reaction at t = 90 min. **(c)** Second ligation at t = 0 min; MaxS2pS11pK31AcK57Ac(1-91) thioester segment (**4's**), hydrolysis of MaxS2pS11pK31AcK57Ac(1-91) thioester (**4's'**), and Cys-Max(93-151)-K segment (**1**). **(d)** Crude ligation reaction at t = 120 min; ligated product (**5'**) **(e)** Crude desulfurization reaction at t = 12 h; desulfurized segment **1** (**1'**). **(f)** RP-HPLC purified **MaxS2pS11pK31AcK57Ac** with the observed mass 17520.2 ± 1.0 Da, calculated mass 17518.8 Da (average isotopes). The UV absorbance was monitored at 214 nm and the m/z data was acquired over the marked region in the chromatogram.

5.3 Chemical synthesis of MaxS2pS11p and MaxK31AcK57Ac

The synthesis of **MaxS2pS11p** and **MaxK31AcK57Ac** was carried out as previously reported.[7,8] The analytical LC-MS of the final products are shown below.

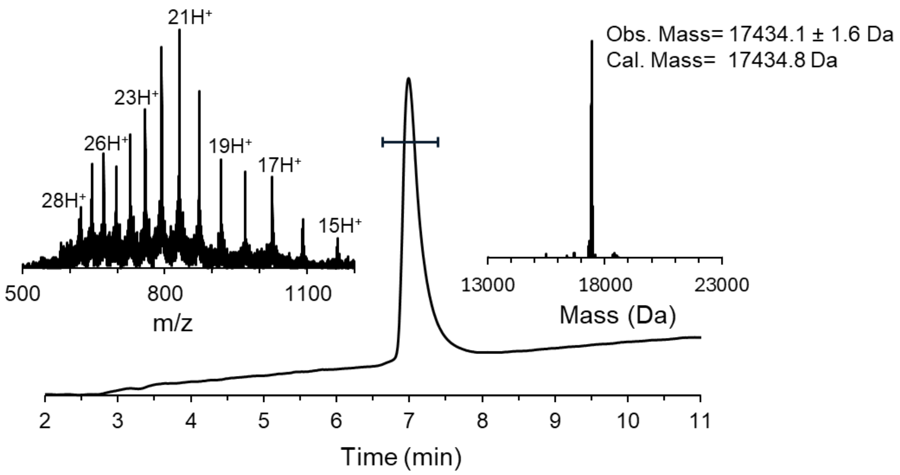

Figure S8. Analytical LC-MS of purified **MaxS2pS11p** with the observed mass 17434.1 ± 1.6 Da, calculated mass 17434.8 Da (average isotopes). The UV absorbance was monitored at 214 nm and the m/z data was acquired over the marked region in the chromatogram. LC-MS analysis was carried out with Method A depicted in section 1.2.

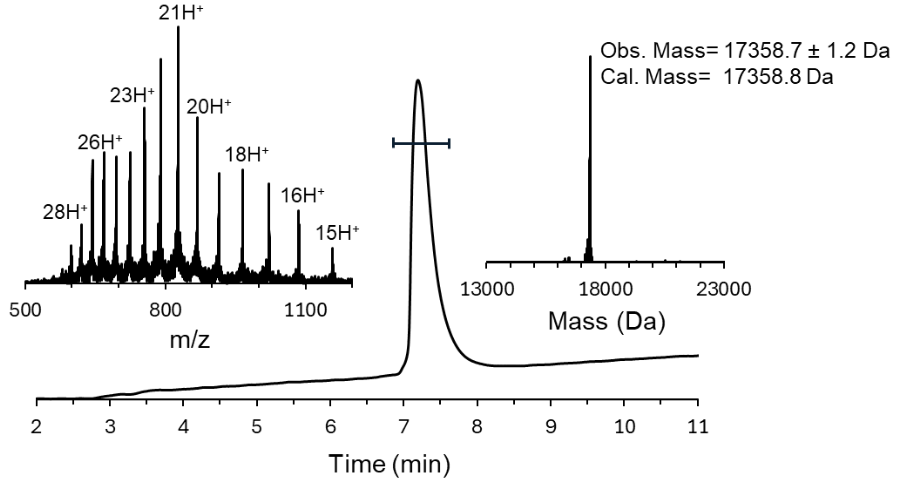

Figure S9. Analytical LCMS of purified **MaxK31AcK57Ac** with the observed mass 17358.7 ± 1.2 Da, calculated mass 17358.8 Da (average isotopes). The UV absorbance was monitored at 214 nm and the m/z data was acquired over the marked region in the chromatogram. LC-MS analysis was carried out with Method A depicted in section 1.2.

6. Folding of Max variants

Synthetic Max variants were dissolved in DMSO (2.5 mM) and then diluted into 10 mM MES, 150 mM KCl, 1 mM $MgCl_2$, 10% glycerol buffer (pH 6) using an Amicon® Ultra- 0.5 mL 3K MWCO spin filtration unit to provide the desired Max variants in 50 µM final concentration in 10 mM MES, 150 mM KCl, 1 mM $MgCl_2$, 10% glycerol buffer (pH 6). The concentrations of Max analogs were determined using a NanoDrop ND-1000 spectrophotometer.

7. Circular Dichroism (CD) Analysis

CD analysis was performed using a Chirascan circular dichroism spectrometer with a 0.1 mm path-length quartz cuvette. Max variants were prepared at a concentration of 10 µM in 50.0 µL of 10 mM MES, 150 mM KCl, 1 mM $MgCl_2$, and 10% glycerol buffer (pH 6.0). CD spectra of all samples were recorded at 20 °C from 190 nm to 270 nm in a 1.0 nm step with 3.0 nm slit bandwidth and an averaging time of three seconds per wavelength. For DNA-binding studies, an annealed E-box DNA probe (10.0 µM) was prepared in the same buffer. A sample containing 5 µM DNA and protein (1:1) in a final volume of 50.0 µL was then prepared. CD spectra of each complex were recorded under identical conditions.

All experiments were performed in triplicate.

The helical percentage (H_α) was calculated from the Luo-Baldwin formula.[9]

$$H_\alpha\ (\%) = (\theta_{222}\ \text{nm}-\theta c)/(\theta\infty_{222}\text{nm}-\theta c)$$

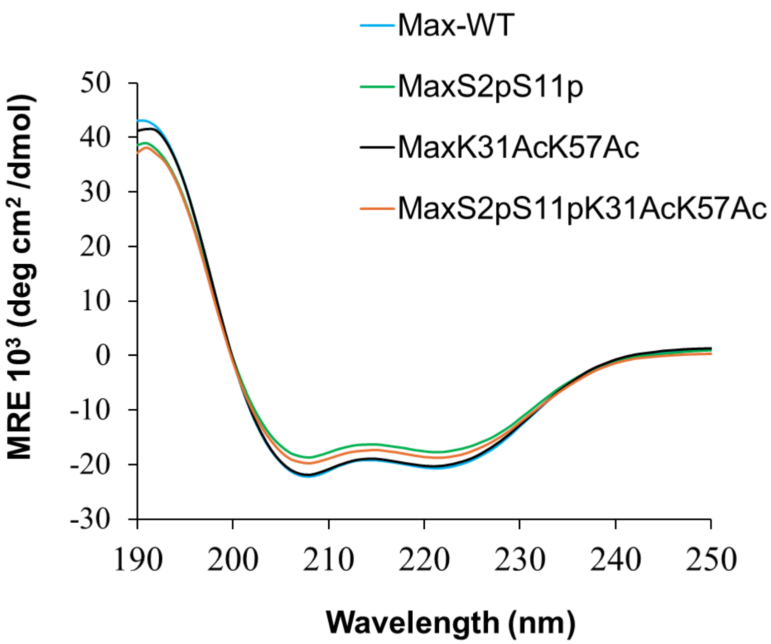
Max-WT
MaxS2pS11p
MaxK31AcK57Ac
MaxS2pS11pK31AcK57Ac
MRE 10³ (deg cm² /dmol)
Wavelength (nm)

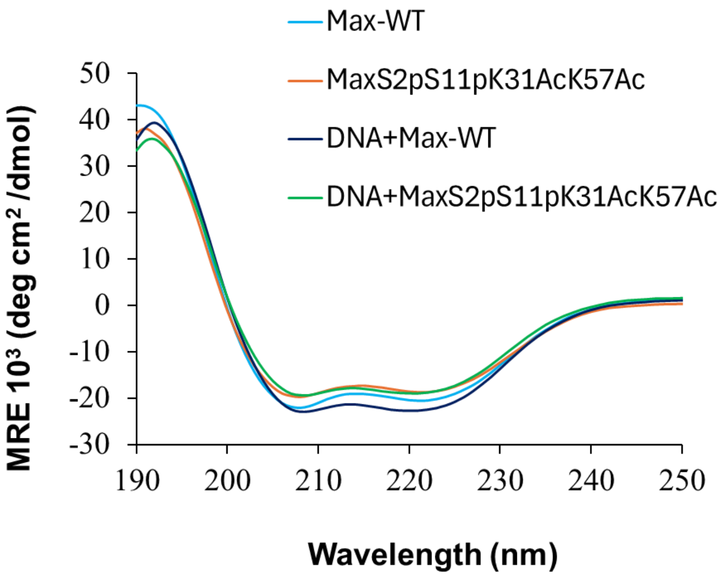
Max-WT
MaxS2pS11pK31AcK57Ac
DNA+Max-WT
DNA+MaxS2pS11pK31AcK57Ac
MRE 10³ (deg cm² /dmol)
Wavelength (nm)

8. DNA-Binding Analysis and Electrophoretic Mobility-Shift Assay (EMSA)

An E-box DNA probe (4.0 µL, 10.0 µM) was added to 0.6 mL Eppendorf tubes containing 36.0 µL, 34.0 µL, 32.0 µL, 30.0 µL, or 28.0 µL of 10 mM MES, 150 mM KCl, 1 mM $MgCl_2$, and 10% glycerol buffer (pH 6). The target protein analog, prepared in the same buffer, was then added at volumes of 2.0 µL, 4.0 µL, 6.0 µL, or 8.0 µL (40.0 µM) to tubes containing 34.0 µL, 32.0 µL, 30.0 µL, or 28.0 µL of buffer and 4 µL of E-box DNA probe, respectively. The final concentrations in the mixture were 1.0 µM for DNA and either 0 µM, 2.0 µM, 4.0 µM, 6.0 µM, or 8.0 µM for the protein. Each tube was thoroughly mixed by pipetting up and down and then incubated at room temperature for 30 min. Concurrently, an 8% TBE gel (1.0 mm thick, 10 wells) was prepared. After incubation, the DNA-binding activity of each protein analog was evaluated by EMSA. For this assay, 5.0 µL of the DNA-protein mixture was mixed with 1.0 µL of DNA Loading Dye (6X). 5.0 µL of this mixture was then loaded onto the 8% TBE polyacrylamide gel and electrophoresed at 90V for 40 min. Following electrophoresis, the gel was washed three times with water (30 seconds each) and stained with Ethidium Bromide in 1x TBE buffer for 15 min at room temperature. Bands representing bound and unbound DNA were visualized using an A2S Vilber Fusion FX imager.

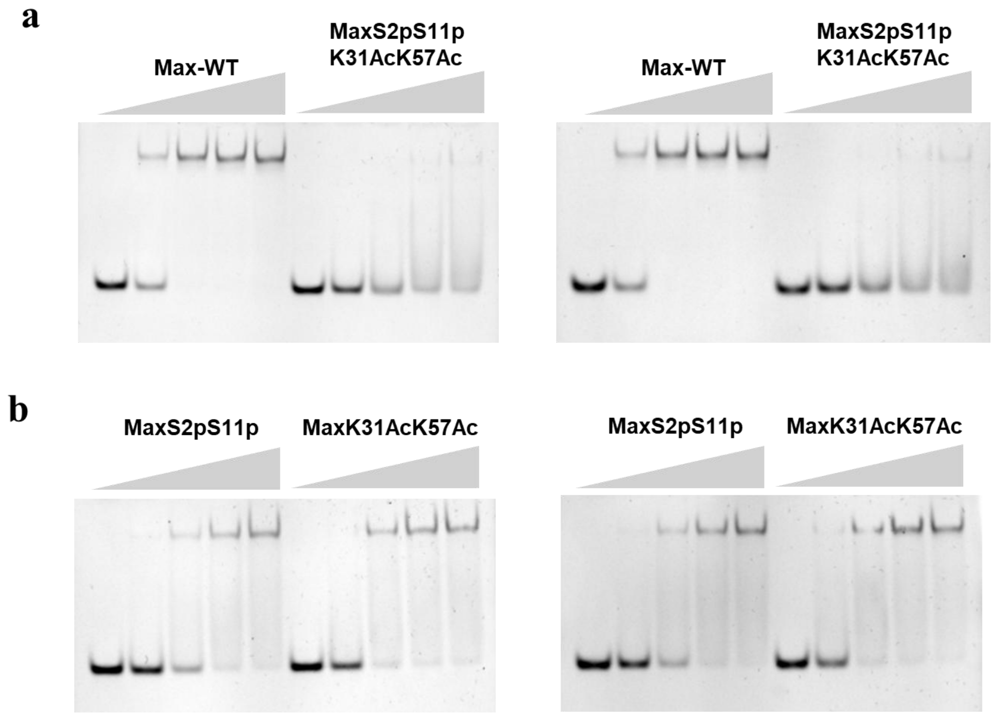

Figure S10. EMSA experiment of Max analogs along with replicate. **(a).** EMSA experiment of **Max-WT** and **MaxS2pS11pK31AcK57Ac** analogs along with replicate. Conditions: 1 µM DNA probe and 0 µM, 2 µM, 4 µM, 6 µM, and 8 µM protein. **(b).** EMSA experiment of **MaxS2pS11p** and **MaxK31AcK57Ac** analogs along with replicate. Conditions: 1 µM DNA probe and 0 µM, 2 µM, 4 µM, 6 µM, and 8 µM protein.

9. Size Exclusion Chromatography (SEC) Analysis

The folded Max analogs **Max-WT** and **MaxS2pS11pK31AcK57Ac** were characterized via Size Exclusion Chromatography (SEC) using ÄKTA Pure on a Superdex 75 column. The column was equilibrated with a buffer consisting of 10 mM MES, 500 mM KCl, 100 mM NaCl and 1 mM $MgCl_2$. Both Max variants were eluted as a single peak at around 9 ml, which corresponds to the mass of a Max homodimer (~35 kDa).

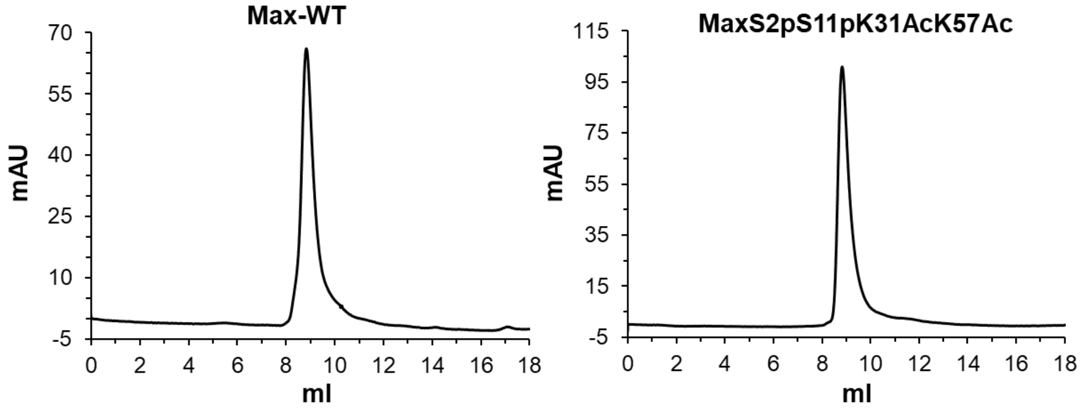

Figure S11. The size exclusion chromatography (SEC) experiments show both Max variants are homogeneous species with a molecular weight corresponding to Max homodimers (35 kDa). The UV absorbance was monitored at 220 nm.

10. Octet BioLayer Interferometry (BLI) Binding Assay

10.1 Max-WT BLI binding assay

Biolayer interferometry (BLI) assay was performed using an Octet Red R4 System (ForteBio; Menlo Park,CA) in 96 well plates. Streptavidin Octet biosensors (ForteBio; Menlo Park, CA) were dipped into 200 µL of 0.1% BSA, 0.02% Tween-20, 1x PBS (kinetic buffer) for 10 min and then 60 more seconds in the kinetic buffer to obtain the baseline. Then the sensors were dipped into 200 µL 65 nM of biotinylated E-box DNA probe in the kinetic buffer for the loading step (300 sec). Sensors were then dipped into the kinetic buffer for 120 sec. Next, the tips were loaded with **Max-WT** prepared in kinetic buffer at the indicated concentrations for 210 seconds to obtain the association curve. Then the tips were dipped into the kinetic buffer for 480 seconds to obtain the dissociation curve. Measurements were carried out at 25 °C. Data was analyzed using the ForteBio Data Analysis software. The association and dissociation curves are fitted with Fortebio Biosystems with a 1:1 Binding Model (global fitting algorithm) to obtain the K_D.

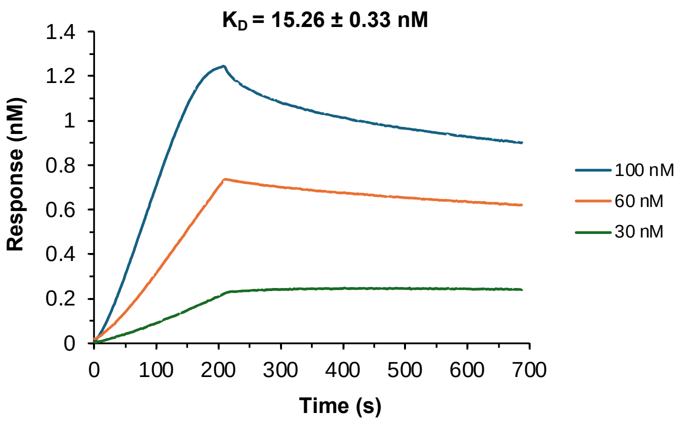

Figure S12. Sensorgrams from BLI analysis of the binding of **Max-WT** to the E-box DNA probe.

10.2 MaxS2pS11pK31AcK57Ac BLI binding assay

Biolayer interferometry (BLI) assay was performed using an Octet Red R4 System (ForteBio; Menlo Park,CA) in 96 well plates. Streptavidin Octet biosensors (ForteBio; Menlo Park, CA) were dipped into 200 µL of 0.1% BSA, 0.02% Tween-20, 1x PBS (kinetic buffer) for 10 min and then 60 more seconds in the kinetic buffer to obtain the baseline. Then the sensors were dipped into 200 µL 65 nM of biotinylated E-box DNA probe in the kinetic buffer for the loading step (300 sec). Sensors were then dipped into the kinetic buffer for 120 sec. Next, the tips were loaded with **MaxS2pS11pK31AcK57Ac** prepared in kinetic buffer at the indicated concentrations for 600 seconds to obtain the association curve. Then the tips were dipped into the kinetic buffer for 90 seconds to obtain the dissociation curve. Measurements were carried out at 25 °C. Data was analyzed using the ForteBio Data Analysis software. The association and dissociation curves are fitted with Fortebio Biosystems with a 1:1 Binding Model (global fitting algorithm) to obtain the K_D.

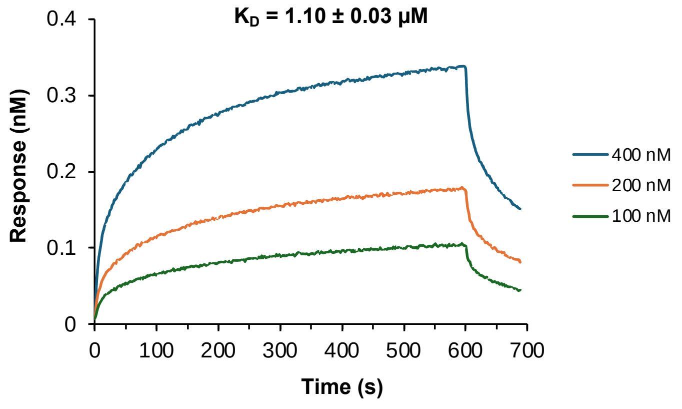

Figure S13. Sensorgrams from BLI analysis of the binding of **MaxS2pS11pK31AcK57Ac** to the E-box DNA probe.

11. References

(1) Zheng, J.-S.; Tang, S.; Qi, Y.-K.; Wang, Z.-P.; Liu, L. Chemical Synthesis of Proteins Using Peptide Hydrazides as Thioester Surrogates. *Nat Protoc* **2013**, *8* (12), 2483–2495.

(2) Ralhan, K.; KrishnaKumar, V. G.; Gupta, S. Piperazine and DBU: A Safer Alternative for Rapid and Efficient Fmoc Deprotection in Solid Phase Peptide Synthesis. *RSC Adv* **2015**, *5* (126), 104417–104425.

(3) Dawson, P. E.; Muir, T. W.; Clark-Lewis, I.; Kent, S. B. H. Synthesis of Proteins by Native Chemical Ligation. *Science (1979)* **1994**, *266* (5186), 776–779.

(4) Fang, G.; Li, Y.; Shen, F.; Huang, Y.; Li, J.; Lin, Y.; Cui, H.; Liu, L. Protein Chemical Synthesis by Ligation of Peptide Hydrazides. *Angewandte Chemie International Edition* **2011**, *50* (33), 7645–7649.

(5) Wan, Q.; Danishefsky, S. J. Free-Radical-Based, Specific Desulfurization of Cysteine: A Powerful Advance in the Synthesis of Polypeptides and Glycopolypeptides. *Angewandte Chemie International Edition* **2007**, *46* (48), 9248–9252.

(6) Haase, C.; Rohde, H.; Seitz, O. Native Chemical Ligation at Valine. *Angewandte Chemie International Edition* **2008**, *47* (36), 6807–6810.

(7) Nithun, R. V.; Yao, Y. M.; Lin, X.; Habiballah, S.; Afek, A.; Jbara, M. Deciphering the Role of the Ser-Phosphorylation Pattern on the DNA-Binding Activity of Max Transcription Factor Using Chemical Protein Synthesis. *Angewandte Chemie* **2023**, *135* (47).

(8) Nithun, R. V.; Yao, Y. M.; Harel, O.; Habiballah, S.; Afek, A.; Jbara, M. Site-Specific Acetylation of the Transcription Factor Protein Max Modulates Its DNA Binding Activity. *ACS Cent Sci* **2024**, *10* (6), 1295–1303.

(9) Shepherd, N. E.; Hoang, H. N.; Abbenante, G.; Fairlie, D. P. Single Turn Peptide Alpha Helices with Exceptional Stability in Water. *J Am Chem Soc* **2005**, *127* (9), 2974–2983.
